# Cholesterol-binding protein TSPO2 coordinates maturation and proliferation of terminally differentiating erythroblasts

**DOI:** 10.1074/jbc.RA119.011679

**Published:** 2020-05-01

**Authors:** Benjaporn Kiatpakdee, Kota Sato, Yayoi Otsuka, Nobuto Arashiki, Yuqi Chen, Takuya Tsumita, Wataru Otsu, Akito Yamamoto, Reo Kawata, Jumpei Yamazaki, Yoshikazu Sugimoto, Kensuke Takada, Narla Mohandas, Mutsumi Inaba

**Affiliations:** 1 Laboratory of Molecular Medicine, Graduate School of Veterinary Medicine, Hokkaido University, Sapporo, Japan; 2 Shirakawa Institute of Animal Genetics, Shirakawa, Japan; 3 Red Cell Physiology Laboratory, New York Blood Center, New York, New York, USA

**Keywords:** cholesterol, cytokinesis, cell cycle, erythropoiesis, erythrocyte, Na+/K+-ATPase, erythroblast, TSPO2

## Abstract

TSPO2 (translocator protein 2) is a transmembrane protein specifically expressed in late erythroblasts and has been postulated to mediate intracellular redistribution of cholesterol. We identified *TSPO2* as the causative gene for the HK (high-K^+^) trait with immature red cell phenotypes in dogs and investigated the effects of the TSPO2 defects on erythropoiesis in HK dogs with the *TSPO2* mutation and *Tspo2* knockout (*Tspo2*^−/−^) mouse models. Bone marrow–derived erythroblasts from HK dogs showed increased binucleated and apoptotic cells at various stages of maturation and shed large nuclei with incomplete condensation when cultured in the presence of erythropoietin, indicating impaired maturation and cytokinesis. The canine TSPO2 induces cholesterol accumulation in the endoplasmic reticulum and could thereby regulate cholesterol availability by changing intracellular cholesterol distribution in erythroblasts. *Tspo2*^−/−^ mice consistently showed impaired cytokinesis with increased binucleated erythroblasts, resulting in compensated anemia, and their red cell membranes had increased Na,K-ATPase, resembling the HK phenotype in dogs. *Tspo2*-deficient mouse embryonic stem cell–derived erythroid progenitor (MEDEP) cells exhibited similar morphological defects associated with a cell-cycle arrest at the G_2_/M phase, resulting in decreased cell proliferation and had a depletion in intracellular unesterified and esterified cholesterol. When the terminal maturation was induced, *Tspo2*^−/−^ MEDEP cells showed delays in hemoglobinization; maturation-associated phenotypic changes in CD44, CD71, and TER119 expression; and cell-cycle progression. Taken together, these findings imply that TSPO2 is essential for coordination of maturation and proliferation of erythroblasts during normal erythropoiesis.

## Introduction

Erythropoiesis is an essential process that produces sufficient numbers of the enucleate red blood cell (RBC) from the erythroid precursor cell. This process proceeds through inexorably linked terminal maturation and cell proliferation of erythroblasts and is tightly regulated by the cooperation among mitogenic, differentiating, and antiapoptotic factors (1, 2). Recent advances as well as earlier studies have defined the mechanisms for various changes during erythropoiesis, including nuclear condensation (3), enucleation (4, 5), expulsion of organelles (6), and plasma membrane remodeling (7–9). These findings are directly relevant to pathobiology of various anemias, precise evaluation of different hematologic conditions, and improvement of *ex vivo* production of functional RBCs for therapeutics. However, the factors implicated in regulation of maturation and proliferation in erythroblasts are yet to be fully defined, although previous studies have documented several genetic factors that determine the RBC traits in humans (10).

Cation contents in mature RBCs (erythrocytes) are quite different among species (11). Human and rodent erythrocytes possess high Na,K-ATPase activity, resulting in high intracellular K^+^ concentration (HK RBCs). In contrast, canine erythrocytes have low K^+^ concentration (LK RBCs) because of total loss of Na,K-ATPase during reticulocyte maturation into erythrocytes (12, 13). However, some dogs possess HK RBCs because they retain Na,K-ATPase in their erythrocytes (12, 14–16). This HK phenotype, an autosomal recessive trait, is accompanied with various characteristics of precursor cells, including the persistence of immature-type glycolytic isozymes and increased energy consumption (17, 18). Hence, the HK RBC phenotype likely represents an impaired regulation in orderly maturation of erythroblasts, and the molecular basis of the HK trait would provide clues to some aspects of erythropoiesis.

Here, we first report identification of the mutations in the translocator protein 2 (TSPO2) gene as the molecular cause for HK RBC trait based on genome-wide linkage analysis. *TSPO2* has been recognized as a paralogue of *TSPO* (19). TSPO is a five-membrane–spanning protein that is localized primarily in the outer mitochondrial membrane and is ubiquitously expressed in various tissues. TSPO has been implicated in various cellular processes, including cholesterol and heme transport, steroidogenesis, mitochondrial respiration, apoptosis, and cell proliferation (20, 21). In contrast to TSPO, TSPO2 shows erythroid-specific expression and localization at the endoplasmic reticulum (ER), nuclear, and plasma membranes (19, 22). It has the ability to bind cholesterol and is involved in cholesterol redistribution during erythropoiesis (19). Intriguingly, impaired reticulocyte maturation due to markedly increased cellular cholesterol (6) and a role for lipid raft assembly with GTPases and F-actin in enucleation (23) indicate the importance of cholesterol homeostasis. Further, hypocholesterolemia in patients of chronic anemias suggests increased cholesterol requirements for erythroid cell expansion (24). However, the roles of cholesterol metabolism in regulating erythropoiesis have not been fully defined.

Based on unexpected finding that the HK trait is associated with the *TSPO2* mutations, we examined erythropoiesis in HK dogs and found morphological abnormalities in maturing erythroblasts. To further investigate the roles of TSPO2 in erythropoiesis, we analyzed the effects of *Tspo2* on erythropoiesis in mice and in a murine erythroid precursor cell line, MEDEP-BRC5 (25), which exhibited terminal differentiation most similar to primary murine erythroid cells among several murine erythroid cell lines (26). Our findings demonstrate that TSPO2 function is essential in coordination of erythroblast maturation, cell-cycle progression, cytokinesis, and cell proliferation to ensure efficient erythropoiesis.

## Results

### TSPO2 gene mutations as the cause of the HK trait in dogs

**Figure 1.**
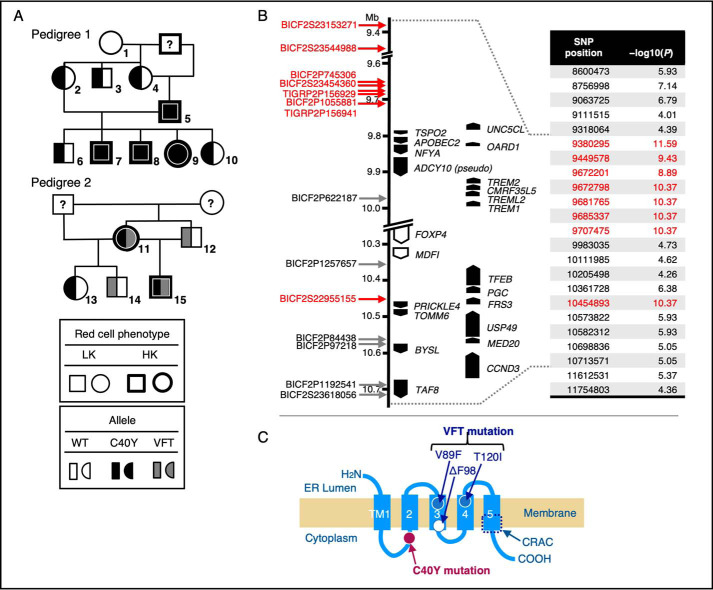
**Identification of the *TSPO2* mutations as the molecular basis for the HK RBC trait in dogs.** *A*, pedigrees of two independent families of Japanese mongrel dogs including six HK and nine LK dogs (a mixed breed of Shiba, numbered 1–15) used in the present study are shown. Another dog with HK RBCs out of these families (purebred Shiba) and eight beagles with LK RBCs were also used in this study. *B*, SNP markers and candidate genes in a region of canine chromosome 12 (9.4–10.7 Mb). Whole-genome genotyping demonstrated that SNP markers shown in *red* had significant association with the HK trait (*p* = 2.59 × 10^−12^ to 4.27 × 10^−11^, indicated as −log_10_(*p*) values). Sequencing of all exons of the 20 annotated genes in this region (indicated by *black arrows*) was carried out for HK and LK dogs. *C*, mutation positions for C40Y and VFT (V89F, ΔF98, and T120I) in cTSPO2 are illustrated on the predicted secondary structure of TSPO2. A cholesterol-binding region (cholesterol recognition amino acid consensus (*CRAC*)) is indicated.

Genome-wide linkage analysis was conducted on seven HK and 17 LK dogs, including 15 dogs from two independent families of Japanese mongrel dogs (Fig. 1*A*). The analysis showed that SNP markers in a region of chromosome 12 (9.4–10.7 Mb), encompassing 22 annotated genes, were significantly associated with the HK trait (*p* = 2.59 × 10^−12^ to 4.27 × 10^−11^). We sequenced all exons for the 20 expressed genes localized in this region for HK and LK dogs and found that only the TSPO2 gene (*TSPO2*) contained significant mutations, namely C40Y and VFT (Fig. 1). No mutations that altered amino acid sequences of the proteins were found to be associated with the HK trait in the exons from other 19 genes examined. The C40Y mutation completely co-segregated with the HK phenotype, and individuals homozygous for C40Y mutation exhibited the HK phenotype in pedigree 1 (Fig. 1*A*). In pedigree 2, there were no HK dogs homozygous for C40Y or VFT mutations, and VFT heterozygotes (No. 12 and No. 14) had the LK phenotype, whereas two HK dogs (No. 11 and No. 15) were compound heterozygotes of C40Y and VFT alleles. These data demonstrate that autosomal recessively inherited C40Y and VFT mutations in *TSPO2* are independent molecular causes for the HK trait in dogs (14, 15).

**Figure 2.**
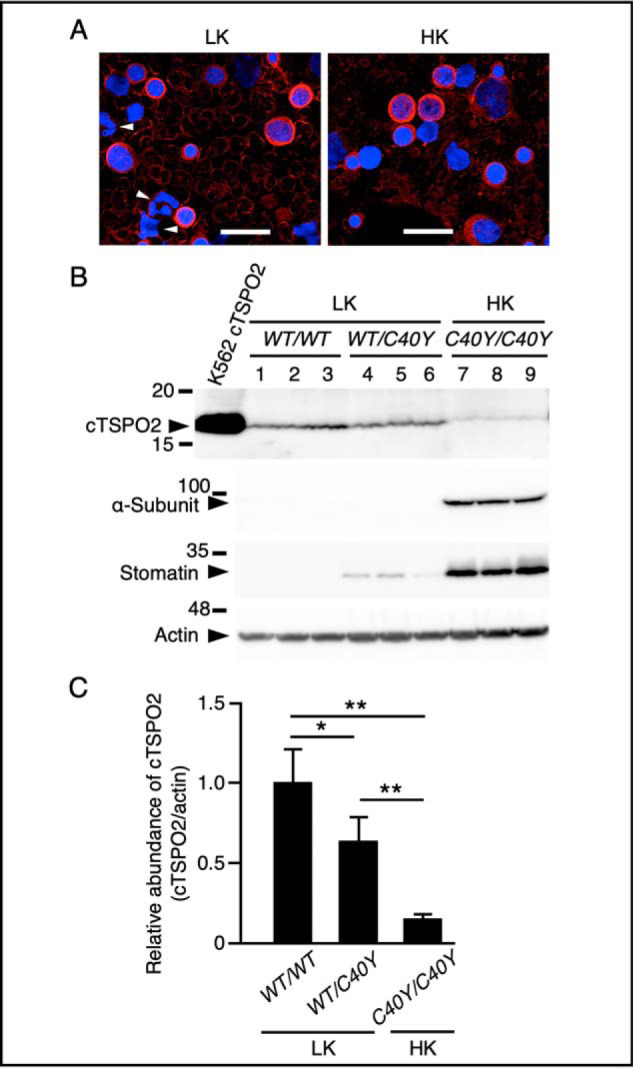
**Canine TSPO2 in BM cells and RBCs.** *A*, BM cells from LK (*LK*, *WT/WT* homozygote) and HK (*HK*, *C40Y/C40Y* homozygote) dogs were reacted with the anti-cTSPO2 followed by staining with secondary antibodies and 4′,6-diamidino-2-phenylindole. The cells with granulocytic nuclei are indicated by *arrowheads. Bars*, 20 μm. *B*, RBC membrane proteins from LK (*LK*) and HK (*HK*) dogs were separated by SDS-PAGE on 10% gels followed by immunoblotting with anti-cTSPO2 (*cTSPO2*; 16 kDa), anti-Na,K-ATPase α-subunit (α-*Subunit*; 96 kDa), anti-stomatin (*Stomatin*; 31 kDa), and anti-actin (*Actin*; 43 kDa) antibodies, respectively. Membranes were prepared from six LK dogs (three each of the *WT/WT* and *WT/C40Y* dogs) and three HK dogs (*C40Y/C40Y*). Each *lane* contained 55 μg (*cTSPO2*) or 10 μg (others) of proteins. Cell extract from K562 cells stably expressing the WT cTSPO2 was loaded as the control (*K562 cTSPO2*). The migrating positions of the size markers are shown in kDa. *C*, signal intensities of cTSPO2 in *B* were analyzed by densitometric scanning and shown as relative values normalized with those of actin. Data are expressed as the means ± S.D. (*error bars*) (*n* = 3). *, *p* < 0.05; **, *p* < 0.01.

TSPO2 was previously shown to be highly expressed in late stage erythroblasts and mediate intracellular trafficking or redistribution of cholesterol (19). Indeed, immunofluorescent microscopy of canine bone marrow (BM) cells with anti-cTSPO2 antibody showed that the fluorescent signals of cTSPO2 are found in the cytoplasm of some of the cells with spherical nuclei, presumably erythroid-lineage cells, but not in granulocytic cells (Fig. 2*A*). Staining of RBC membranes was also apparent.

Immunoblot analysis showed that the anti-cTSPO2 antibody reacted with the 16-kDa cTSPO2 polypeptide in RBC membranes from both LK (homozygous for the WT (*WT/WT*) and heterozygous for WT and C40Y mutation (*WT/C40Y*)) and HK (homozygous for C40Y mutation (*C40Y/C40Y*)) dogs (Fig. 2*B*). Quantification of the relative abundance of cTSPO2 by densitometry scanning demonstrated that the cTSPO2 levels in *WT/C40Y* and *C40Y/C40Y* (HK) RBCs were ∼63 and 15%, respectively, of the mean levels of expression in the *WT/WT* cells (Fig. 2*C*). Na,K-ATPase α-subunit was detectable only in HK RBCs, and a raft-associated protein, stomatin, was most abundant in HK RBCs and was detectable in *WT/C40Y* but not in *WT/WT* cells, consistent with our previous data (12, 13).

### TSPO2 gene mutations impair the function of TSPO2 in transfected cells

**Figure 3.**
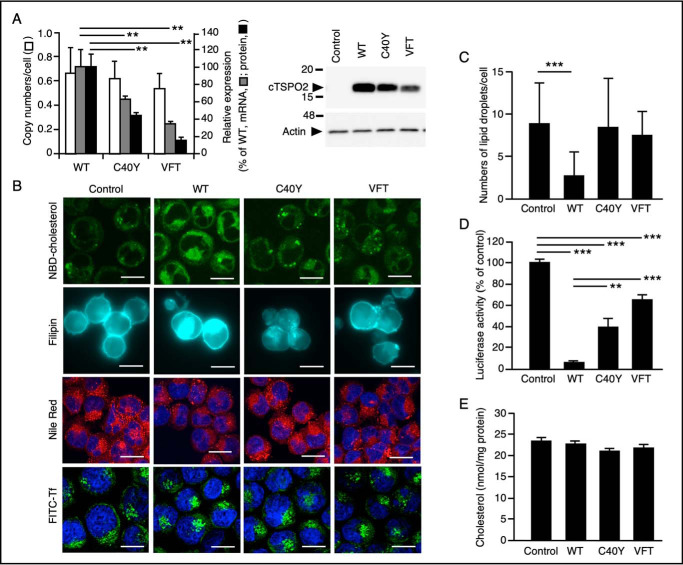
**TSPO2 mutations causative of the HK RBC phenotype are detrimental to the TSPO2 function.** *A*, copy numbers of transfected cTSPO2 cDNAs (indicated by *open bars*) and the contents of cTSPO2 mRNA (indicated by *gray bars*) in stably transfected K562 cells were estimated by quantitative PCR and quantitative RT-PCR, respectively, for the WT (*WT*), C40Y (*C40Y*), and VFT (*VFT*) cTSPO2. The cellular contents of 16-kDa cTSPO2 were quantitated by densitometric scanning of the immunoblots using the anti-cTSPO2 antibody and normalized with actin (43 kDa; indicated by *black bars*). A representative immunoblot from three independent experiments is shown in the *right panel* (an entire blot is shown in Fig. S1). Data are expressed as the means ± S.D. (*error bars*) (*n* = 3). **, *p* < 0.01. The migrating positions of the size markers are shown in kDa. *B*, fluorescent signals of incorporated NBD-cholesterol (*NBD-cholesterol*), nonesterified cholesterol stained with filipin III (*Filipin*), CEs stained with Nile Red (*Nile Red*), and incorporated FITC-transferrin (*FITC-Tf*) in K562 cells stably transfected with the empty vector (*Control*) and WT (*WT*), C40Y (*C40Y*), or VFT (*VFT*) cTSPO2 cDNA. *Bars*, 20 μm. *C*, lipid droplets stained with Nile Red in *B* were counted, and the numbers of vesicles larger than 1 μm in the major axis were compared. Data are expressed as the means ± S.D. (*error bars*) (*n* = 22–28). ***, *p* < 0.001. *D*, cholesterol accumulation in the ER was assessed by an LDLR-promoter luciferase assay. Activities relative to the control cells are expressed as the means ± S.D. (*n* = 3). **, *p* < 0.01; ***, *p* < 0.001. *E*, total cholesterol contents in the cells are expressed as the means ± S.D. (*n* = 3).

To examine whether C40Y and VFT mutations impaired the function of TSPO2, we analyzed the intracellular cholesterol distribution in K562 cells stably expressing the WT or the mutant cTSPO2. These cell lines integrated the transfected cDNAs at nearly equivalent levels (Fig. 3*A*). However, K562 cells expressing C40Y or VFT mutant had significantly lower levels of expression of both cTSPO2 mRNA and protein (detected as the ∼16-kDa polypeptide in immunoblots) than the WT-expressing cells (Fig. 3*A* and Fig. S1).

The K562 cells expressing WT cTSPO2 exhibited dispersed cytoplasmic incorporation of NBD-cholesterol as reported previously for human TSPO2 (19). In contrast, the cells expressing C40Y or VFT mutant, as well as the empty vector–transfected control cells, showed nonpolar and vesicular distribution of NBD-cholesterol (Fig. 3*B*). Likewise, whereas filipin staining demonstrated massive accumulation of free cholesterol in the cytoplasm and the cell periphery of the WT-expressing cells, weaker fluorescent signals were observed in cells expressing the mutants as well as in control cells. In contrast, vesicular signals of cholesteryl esters (CEs), lipid droplets, detected with Nile Red were less abundant in the WT-expressing cells than in other cell lines (Fig. 3, *B* and *C*). Interestingly, no apparent difference was observed among these cell lines in the uptake of transferrin (Fig. 3*B*), which shares the clathrin-mediated endocytosis pathway with cholesterol for incorporation into the cell (27). These data suggest that cTSPO2 induces specific alterations in intracellular distribution of cholesterol and that the cTSPO2 mutants failed to induce such redistribution.

Moreover, K562 cells expressing WT cTSPO2 showed a marked reduction of ∼93%, compared with control cells, in the reporter luciferase activity (Fig. 3*D*) that was driven by sterol regulatory element–binding protein (SREBP)-mediated low-density lipoprotein receptor (LDLR) promoter (28). This indicated that the expression of the WT cTSPO2 caused an accumulation of cholesterol in the ER, leading to a marked suppression of the SREBP-mediated LDLR promoter activation. Because there were no significant differences in total cholesterol contents among different cell lines (Fig. 3*E*), cholesterol accumulation in the ER in the WT-expressing cells was likely the consequence of change in distribution of intracellular cholesterol. Interestingly, cells expressing the C40Y or VFT mutant elicited reporter luciferase activity of 40 and 63%, respectively, of that in control cells (Fig. 3*D*), demonstrating that ER accumulation of cholesterol in the mutant-transfected cells was significantly reduced. These data suggest that the TSPO2 function involves, in part, an accumulation of cholesterol in the ER and that the C40Y and VFT mutations are detrimental to this function.

### Erythroid cell phenotype in the HK dog

**Figure 4.**
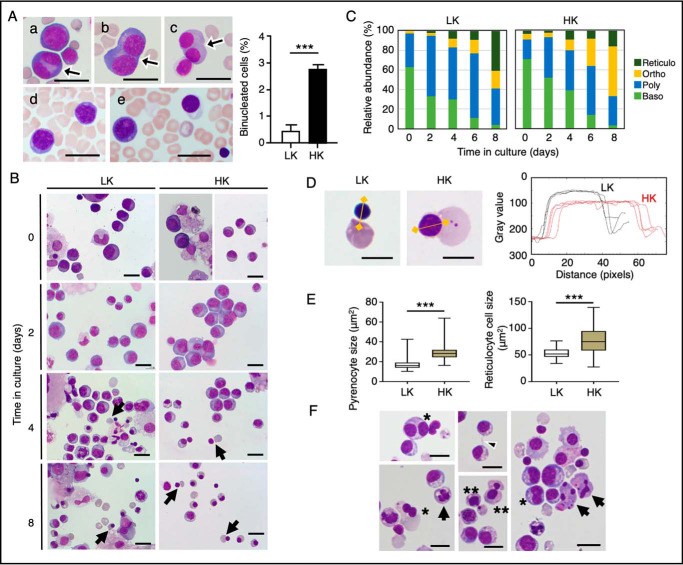
**Abnormal morphologies in erythropoiesis in HK dogs.** *A*, Wright–Giemsa–stained BM aspirate from HK dogs showed increased numbers (shown in the *right panel*) of binucleated erythroblasts (*arrows*) at various stages (*a–c*) compared with LK dogs (*d* and *e*). *Bars*, 20 μm. Data in the *right panel* are expressed as the means ± S.D. (*error bars*) (*n* = 4). ***, *p* < 0.001. *B*, representative Wright–Giemsa–stained BM-derived cells from HK (*HK*) and LK (*LK*) dogs at days 0, 2, 4, and 8 in the second phase of a two-phase liquid culture. Enucleating cells are indicated by *arrows. Bars*, 20 μm. *C*, basophilic erythroblasts (*Baso*), polychromatic erythroblasts (*Poly*), orthochromatic erythroblasts (*Ortho*), and enucleate reticulocytes (*Reticulo*) were counted on Wright–Giemsa–stained smears and shown as a percentage of total erythroid cells. *D* and *E*, enucleating cells on day 4 in the culture were analyzed for the sizes (areas in μm^2^) of expelled pyrenocytes and enucleated reticulocytes. *D*, typical enucleating cells from LK and HK dogs (*left panels*). The *RGB color images* were converted to *gray scale*, and the brightness intensities are shown in *gray values* together with the distance (*right panel*) for the axes exemplified in *yellow lines* in the *left panels. Bars*, 10 μm. ***, *p* < 0.001, *n* = 75. *F*, BM-derived erythroblasts from HK dogs in the second phase of a two-phase liquid culture showed various abnormal morphological features, involving binucleated cells (*asterisks*), long and thin intercellular bridges (*arrowhead*), abnormal cell division (*double asterisks*), and apoptotic appearance (*arrows*). *Bars*, 20 μm.

We studied functional consequences of these *TSPO2* mutations for erythropoiesis. Examination of cells from BM aspirates showed the presence of abundant numbers of binucleated erythroblasts at different developmental states, basophilic, polychromatic, and orthochromatic erythroblasts in HK dogs (homozygotes for C40Y mutation), with their numbers being 7-fold higher than that seen in LK dogs, indicative of a cell-division defect (Fig. 4*A*).

We further characterized the morphological defects of HK erythroblasts during the second phase of two-phase liquid culture, where BM-derived nonadherent mononuclear cells were cultured in the presence of erythropoietin. At days 0 and 2 in culture, basophilic and polychromatic erythroblasts were predominant erythroid cell types in both HK and LK cells (Fig. 4, *B* and *C*). Of note, upon enucleation that was observed on day 3 through day 8, HK orthochromatic erythroblasts shed nuclei with morphologically incomplete chromatin condensation, and the sizes of expelled pyrenocytes and nascent reticulocytes were 1.7- and 1.5-fold, respectively, larger than those of LK cells (Fig. 4, *D* and *E*). Incomplete condensation was also confirmed by the gray values of the nuclei that were decreased by ∼20–30% from those of LK cells on image analysis (Fig. 4*D*, *right*). Moreover, HK cells showed various abnormal morphological features, involving binucleated cells, long intercellular bridges, abnormal cell division, and apoptotic appearance (Fig. 4*E*). These data suggest that the TSPO2 mutations impair maturation and cytokinesis during late stages of terminal erythroid differentiation.

### Erythroid cell maturation in Tspo2 knockout mice

**Figure 5.**
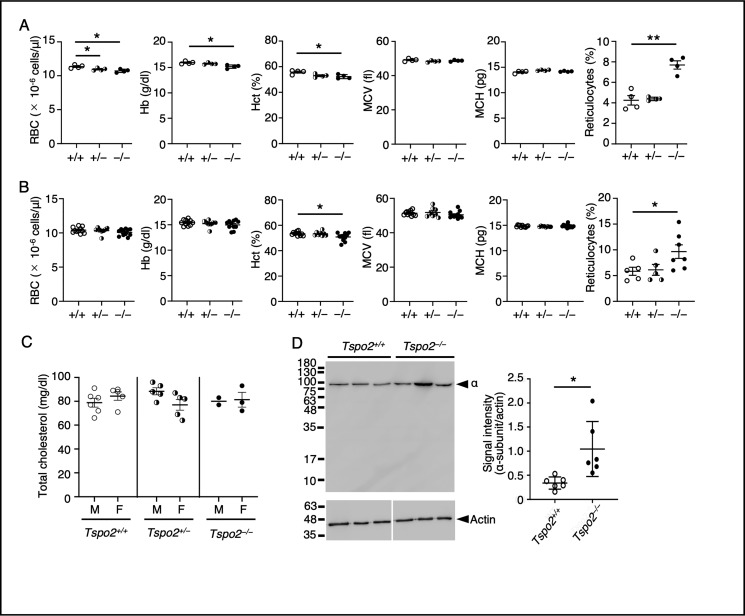
**RBC phenotypes in *Tspo2* knockout mice.** *A*, RBC counts (*RBC*), hemoglobin concentration (*Hb*), hematocrit (*Hct*), mean corpuscular volume (*MCV*), mean corpuscular hemoglobin (*MCH*), and reticulocyte counts (*Reticulocytes*) in peripheral blood of 20-week-old *Tspo2*^+/+^ (+/+, *n* = 4), *Tspo2*^+/−^ (+/−, *n* = 4), and *Tspo2*^−/−^ (−/−, *n* = 4) mice. Data are expressed as the means ± S.E. (*error bars*) *, *p* < 0.05; **, *p* < 0.01. *B*, hematological parameters are shown for *Tspo2*^+/+^ (+/+, *n* = 11), *Tspo2*^+/−^ (+/−, *n* = 8), and *Tspo2*^−/−^ (−/−, *n* = 11) mice at 6–8 weeks of age. Sample numbers for reticulocyte counts are 5, 5, and 7 for +/+, +/−, and −/−, respectively. Data are expressed as the means ± S.E. *, *p* < 0.05. *C*, total cholesterol levels in plasma were determined for *Tspo2*^+/+^ (*n* = 6 for male (*M*) and *n* = 5 for female (*F*)), *Tspo2*^+/−^ (*n* = 5 for both *M* and *F*), and *Tspo2*^−/−^ (*n* = 2 for *M* and *n* = 3 for *F*) mice at ages of 6–8 weeks. Data are expressed as the mean value for two male *Tspo2*^−/−^ mice or the means ± S.E. for others. *D*, RBC membranes were prepared from *Tspo2*^+/+^ and *Tspo2*^−/−^ mice, and the contents of Na,K-ATPase α-subunit were analyzed by immunoblotting as described previously (12). The blot was probed with an antibody to Na,K-ATPase α-subunit (α) and an anti-β-actin antibody to detect β-actin (*Actin*) for the internal control. Each *lane* contained 25 μg of RBC membrane proteins. The abundance of α-subunit relative to actin determined by densitometric scanning was 0.34 ± 0.13 (mean ± S.D. (*error bars*), *n* = 6) in *Tspo2*^+/+^ mice and 1.04 ± 0.57 (mean ± S.D., *n* = 6) in *Tspo2*^−/−^ mice, respectively (*p* < 0.05).

To gain further insight into the role of TSPO2 defect on erythropoiesis, we examined hematopoiesis in germline *Tspo2*-deficient mice (Fig. S2). Adult *Tspo2*^−/−^ mice at 20 weeks of age showed significant reduction in RBC count, hemoglobin concentration, and hematocrit values and a marked increase in reticulocyte count implicating well-compensated mild anemia (Fig. 5*A*). *Tspo2*^+/−^ mice also had a mild but significant reduction in RBC count. However, no remarkable differences were noted in the mean corpuscular volume and the mean corpuscular hemoglobin. *Tspo2*^−/−^ mice at 6–8 weeks of age also showed a significant decrease in hematocrit value and an increase in reticulocyte count (Fig. 5*B*). Because hypocholesterolemia due to increased cholesterol requirements has been demonstrated in patients with chronic anemia (24), we measured the plasma cholesterol levels in *Tspo2*-deficient mice. There was no significant change in total cholesterol levels in *Tspo2*^−/−^ and *Tspo2*^+/−^ mice compared with those in the WT mice (Fig. 5*C*), possibly due to the modest anemic phenotype in these mice.

Na,K-ATPase contents in RBC membranes were higher in *Tspo2*^−/−^ mice than in control *Tspo2*^+/+^ mice (1.04 ± 0.57 *versus* 0.34 ± 0.13 for the relative abundance, mean ± S.D., *n* = 6; Fig. 5*B*), compatible with the HK RBC phenotype shown in Fig. 2 and our previously reported findings (12, 13).

**Figure 6.**
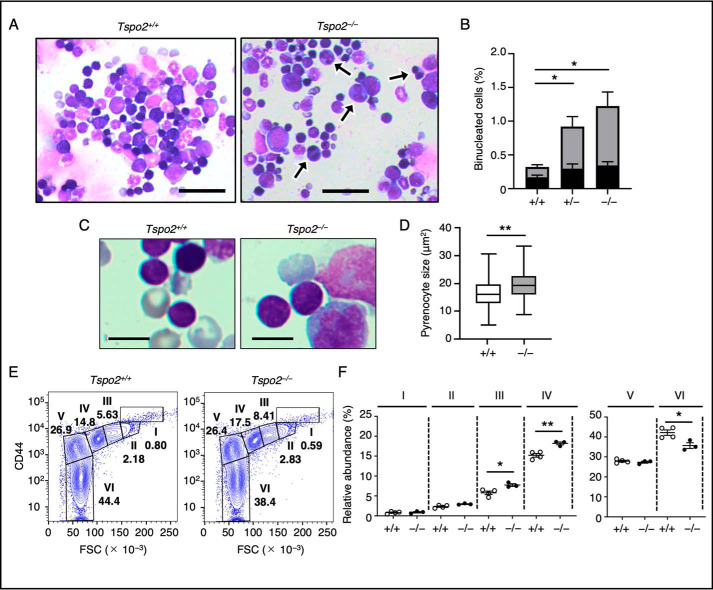
**Erythroid phenotypes in *Tspo2* knockout mice.** *A* and *B*, Wright–Giemsa–stained BM aspirates from 20-week-old *Tspo2*^+/+^ (*Tspo2*^+/+^) and *Tspo2*^−/−^ (*Tspo2*^−/−^) mice showed increased numbers of binucleated erythroblasts (*arrows*) at various stages in *Tspo2*^−/−^ mice. *Bars*, 50 μm. Relative abundance of binucleated erythroblasts are expressed as the means ± S.E. (*error bars*) (*n* = 4) in *B. Columns* in *gray* or *black* indicate the abundance of polychromatic and orthochromatic erythroblasts (*, *p* < 0.05) or basophilic erythroblasts, respectively. *C* and *D*, enucleating cells on Wright–Giemsa–stained BM aspirates from 20-week-old *Tspo2*^+/+^ and *Tspo2*^−/−^ mice were analyzed for the sizes (areas in μm^2^) of expelled pyrenocytes. **, *p* < 0.01, *n* = 200. *Bars*, 10 μm. *E* and *F*, BM-derived erythroid cells from 20-week-old *Tspo2*^+/+^ (+/+, *n* = 4) and *Tspo2*^−/−^ (−/−, *n* = 3) mice were analyzed for their CD44 expression (*CD44*) together with forward scatter (*FSC*) by flow cytometry (29, 30). Relative abundance of the cells in populations I–VI shown in *E* are indicated in *F*. *, *p* < 0.05; **, *p* < 0.01.

In BM cells, there was a marked increase in binucleated erythroblasts in both *Tspo2*^−/−^ and *Tspo2*^+/−^ mice. Polychromatic and orthochromatic erythroblasts constituted the major proportion (∼70%) of binucleated cells (Fig. 6, *A* and *B*). The size of pyrenocytes in *Tspo2*^−/−^ mice was significantly larger than that in *Tspo2*^+/+^ mice (Fig. 6, *C* and *D*). Flow cytometric analysis of CD44 surface expression together with forward scatter for BM-derived erythroid cells from age-matched *Tspo2*^+/+^ and *Tspo2*^−/−^ mice showed similar profiles, and the erythroid cells could be divided into six fractions, as reported previously (29, 30). Compatible with the increase in their binucleated forms, relative abundance of polychromatic and orthochromatic erythroblasts (populations III and IV) was significantly higher in *Tspo2*^−/−^ mice than in WT mice (Fig. 6, *E* and *F*). Indeed, sorted cells in populations I–IV from *Tspo2*^−/−^ mice contained binucleated cells (data not shown).

These hematological and morphologic features in *Tspo2*^−/−^ mice appear very similar to erythroid phenotypes seen in HK dogs and reveal that the *Tspo2* defect predominantly impairs cytokinesis of erythroblasts and causes differentiation defects between the basophilic and orthochromatic erythroblast stages.

### Erythroid cell maturation of Tspo2 knockout cell line

To further understand the effect of the TSPO2 defect on terminal erythropoiesis, we created a *Tspo2*-deficient cell clone of MEDEP-BRC5 cells (*Tspo2*^−/−^ MEDEP cells), which exhibit characteristics of proerythroblasts or CFU-E. These cells can be differentiated into reticulocytes (25), and their terminal differentiation is very similar to that of primary murine erythroid cells (26). Two independent clones of *Tspo2*^−/−^ MEDEP cells (KO13 and KO26) obtained exhibited similar impairments in cell proliferation and hemoglobinization, and further detailed studies were performed using the clone KO13.

**Figure 7.**
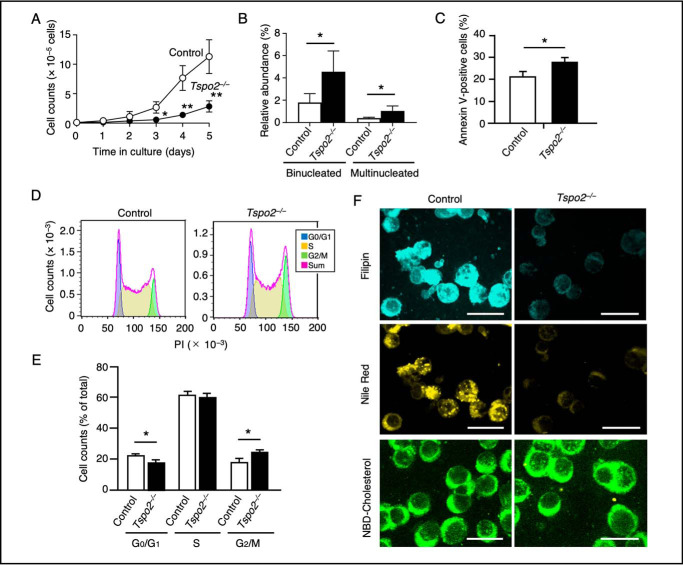
**Morphological and cellular phenotypes in self-renewal of *Tspo2* knockout MEDEP cells.** *A*, proliferation of control (*Control*) and *Tspo2*^−/−^ (*Tspo2*^−/−^) MEDEP cells. Cells were cultured in the absence of erythropoietin. Data are expressed as the means ± S.D. (*error bars*) (*n* = 4). *, *p* < 0.05; **, *p* < 0.01. *B*, after 48 h in culture, cytospin smears were prepared, stained with Wight–Giemsa, and counted for binucleated and multinucleated cells. Data are expressed as the means ± S.D. (*n* = 4). *, *p* < 0.05. *C*, the relative abundance of annexin V-positive apoptotic cells was determined by flow cytometry, and the data are expressed as the means ± S.D. (*n* = 7). *, *p* < 0.05. *D* and *E*, cell-cycle analysis of control and *Tspo2*^−/−^ cells. Cells were collected at 48 h, stained with PI, and subjected to flow cytometry. Relative abundance of G_0_/G_1_, S, and G_2_/M phases was determined using FlowJo software, and representative histograms are shown in *D*. Data shown in *E* are expressed as the means ± S.D. (*n* = 6). *, *p* < 0.05. *F*, control and *Tspo2*^−/−^ MEDEP cells were counterstained with filipin III (*Filipin*) and Nile Red (*Nile Red*) to detect free cholesterol and CEs, respectively. NBD-cholesterol incorporated into the cells after 10 min of incubation (*NBD-cholesterol*) is also shown. *Bars*, 20 μm.

*Tspo2*^−/−^ MEDEP cells revealed reduced *Tspo2* mRNA expression (Fig. S3). Various morphological and cellular defects in self-renewal, including reduced cell proliferation (Fig. 7*A*), increased binucleated and multinucleated cells (Fig. 7*B*), and a 33% increase in annexin V–positive apoptotic cells (Fig. 7*C*) compared with control cells could be documented. These defects in *Tspo2*^−/−^ cells were accompanied by an arrest at G_2_/M stage as demonstrated by the cell number in the G_2_/M population that was increased by 39% compared with control cells (Fig. 7, *D* and *E*). In addition, whereas staining with filipin and Nile Red showed abundant cytoplasmic signals of free cholesterol and CEs, respectively, in control cells, both filipin and Nile Red signals in *Tspo2*^−/−^ cells exhibited profound reductions in staining. In contrast, *Tspo2*^−/−^ cells showed uptake of NBD-cholesterol comparable with that in the control cells (Fig. 7*F*). These data imply that *Tspo2*^−/−^ MEDEP cells had a decreased ability to regulate both free and esterified cholesterol, despite their ability to incorporate extracellular cholesterol into the cells.

**Figure 8.**
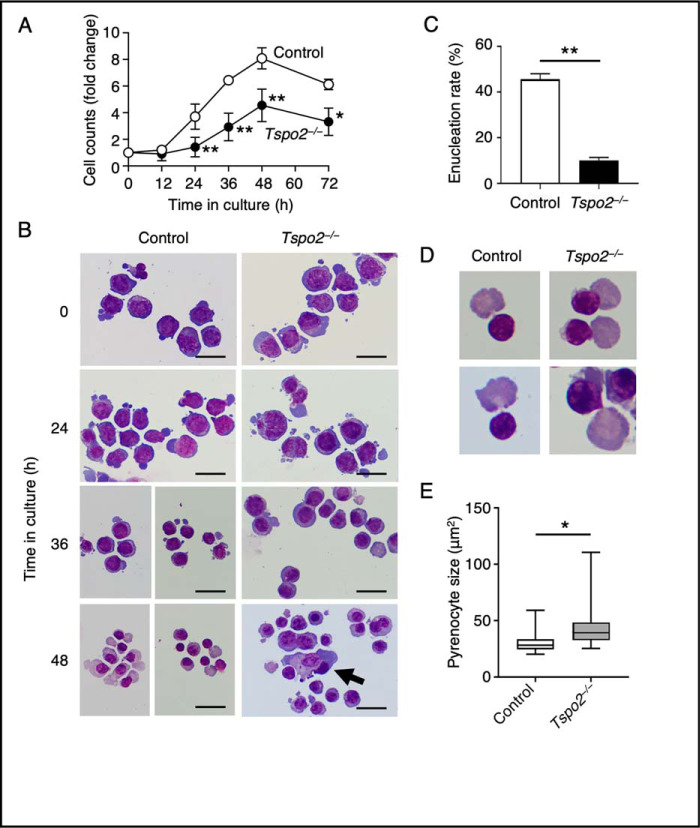
**Impaired proliferation and abnormal morphology in maturing *Tspo2* knockout MEDEP cells.** *A*, control (*Control*) and *Tspo2*^−/−^ (*Tspo2*^−/−^) MEDEP cells were cultured in the presence of erythropoietin to induce erythroid terminal maturation. The changes in cell counts are shown in multiples of the initial cell counts and are expressed as the means ± S.D. (*error bars*) (*n* = 3). *, *p* < 0.05; **, *p* < 0.01. *B*, at the indicated time of incubation, cytospin smears were prepared and stained with Wight–Giemsa. *Tspo2*^−/−^ cell culture contained increased numbers of binucleated cells and the cells with apoptotic features (*arrow*). *Bars*, 20 μm. *C*, at 48 h in culture, enucleating cells in the control and *Tspo2*^−/−^ cell cultures were counted, and their relative abundance against total cells (200 cells) is shown as the enucleation rate. Data are expressed as the means ± S.D. (*n* = 3). **, *p* < 0.01. *D* and *E*, enucleating cells in the control and *Tspo2*^−/−^ cell cultures (*n* = 50 for each; representatives are shown in *D*) were analyzed for the size of extruded pyrenocytes. *, *p* < 0.05.

After induction with erythropoietin, the control cell numbers increased by 8-fold at 48 h, whereas *Tspo2*^−/−^ cells showed only a 4.5-fold increase (Fig. 8*A*). During this period, both control and *Tspo2*^−/−^ MEDEP cells matured progressively and underwent enucleation. After 48 h, the control culture comprised mainly the cells whose morphology resembled that of orthochromatic erythroblasts, enucleated reticulocytes, and pyrenocytes. In contrast, cells in *Tspo2*^−/−^ culture were heterogeneous in appearance, containing cells ranging from basophilic to orthochromatic erythroblasts with considerably increased numbers of binucleated and apoptotic cells (Fig. 8*B*). There were much fewer enucleating cells in *Tspo2*^−/−^ cell culture compared with the control culture (10.0 ± 1.3% *versus* 45.7 ± 2.4%, mean ± S.D., *n* = 3; Fig. 8*C*). The size of pyrenocytes in *Tspo2*^−/−^ cell culture was significantly larger than that in control cell culture (Fig. 8, *D* and *E*).

**Figure 9.**
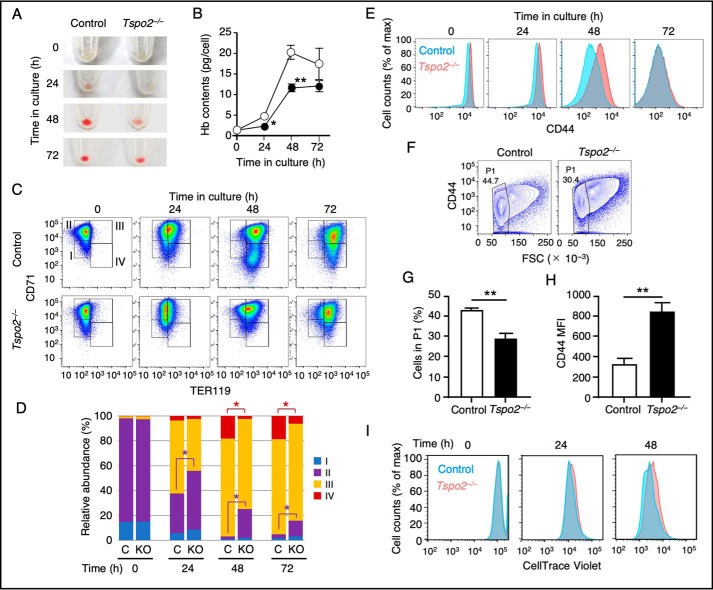
**Impaired maturation in *Tspo2* knockout MEDEP cells.** Control (*Control*) and *Tspo2*^−/−^ (*Tspo2*^−/−^) MEDEP cells were incubated as described in the legend to Fig. 8. *A* and *B*, cells were collected at the indicated time, and the color of cell pellets (*A*) and hemoglobin (*B*) contents was examined. In *B*, data are expressed as the means ± S.D. (*error bars*) (*n* = 3). *, *p* < 0.05; **, *p* < 0.01. *C* and *D*, representative data for CD71 *versus* TER119 in control and *Tspo2*^−/−^ MEDEP cells at the indicated time of incubation (*C*). The cells are divided into four fractions (*I–IV*) as indicated in *C*, and the relative abundance (%) of the cells in each fraction at different time points is shown in *D*. Data are expressed as the mean values (*n* = 3). S.D. values are not shown for simplification. *, *p* < 0.05. In *D*, control and *Tspo2*^−/−^ cells are indicated as *C* and *KO*, respectively. *E–H*, representative data from three independent flow cytometric analyses for cell-surface CD44 *versus* cell counts in control and *Tspo2*^−/−^ cells at the indicated time in culture (*E*) and CD44 *versus* FSC at 48 h (*F*). P1 fractions from control and *Tspo2*^−/−^ cells shown in *F* were analyzed for the abundance relative to the total numbers of cells (*G*) and MFI of CD44 (*H*). Data are expressed as the means ± S.D. (*n* = 3). **, *p* < 0.01. *I*, control and *Tspo2*^−/−^ cells were labeled with a tracer dye CellTrace Violet and chased for the decline of incorporated dye by flow cytometry. Representative data from four independent analyses is shown for the fluorescent intensity *versus* cell counts at 0, 24, and 48 h after labeling.

Maturing *Tspo2*^−/−^ MEDEP cells also showed a remarkable reduction in hemoglobin content that was less than 60% of that in control cells at 48 h (Fig. 9, *A* and *B*). However, there was no major difference through the maturation process in the mRNA levels of the genes relevant to hemoglobin synthesis, such as *Hba*, *Hbb*, *TfR*, and *Alas2*, as well as several other genes essential for the terminal erythropoiesis, such as *Gata1*, *Klf1*, *Setd8*, and *Slc4a1*, between *Tspo2*^−/−^ and control cells (Fig. S3).

Furthermore, we found marked differences in the expression of phenotypic markers of erythropoiesis following *Tspo2* knockout. The major population of control and *Tspo2*^−/−^ cells had high CD71 and low TER119 expression levels (CD71^high^/TER119^low^) before induction. Control cells progressed into CD71^high^/TER119^high^ cells after 24 h, and a subpopulation of cells matured into CD71^low^/TER119^high^ cells at 48 h. In contrast, significant numbers of *Tspo2*^−/−^ cells remained in the CD71^high^/TER119^low^ population at 24 h and in the CD71^high^/TER119^high^ population with no increase of CD71^low^/TER119^high^ cells after 48 h (Fig. 9, *C* and *D*). At 72 h, although both control and *Tspo2*^−/−^ cells showed a further reduction in CD71 expression, the CD71^low^/TER119^high^ population was retained at a low level in *Tspo2*^−/−^ cells. Moreover, the median fluorescent intensity (MFI) of CD44 in *Tspo2*^−/−^ MEDEP cells, that was slightly higher than that in control cells at the beginning of culture, remained at higher levels compared with control cells at 48 h and then decreased to levels compatible with control cells at 72 h (Fig. 9*E*). At 48 h, cells could be divided into two populations according to their sizes (P1 fraction and others; Fig. 9*F*). The population of more mature cells with smaller sizes (P1 fraction) in *Tspo2*^−/−^ cells was less abundant compared with that in control cells (29.3 ± 2.6% *versus* 43.6 ± 1.0%, mean ± S.D., *n* = 3; Fig. 9*G*). In addition, the MFI of CD44 in P1 fraction of *Tspo2*^−/−^ cells was significantly higher than that in control cells (840 ± 95 *versus* 323 ± 62, mean ± S.D., *n* = 3; Fig. 9*H*). These data indicate that morphologic and phenotypic maturation of *Tspo2*^−/−^ cells is markedly delayed.

These delays in morphologic maturation, hemoglobinization, and changes in phenotypic markers suggest delayed terminal erythroid differentiation of *Tspo2*^−/−^ cells. To test this, we labeled the cells with the tracer dye CellTrace Violet and analyzed the reduction in tracer dye intensities. The profile for the reduction in tracer dye intensities suggested that *Tspo2*^−/−^ cells slightly lagged behind the control cells in cell division (Fig. 9*I*). In *Tspo2*^−/−^ cells, therefore, the alteration in the cell-cycle progression but not the number of cell divisions is likely to account for the delayed terminal maturation.

Together with an increase in apoptotic cells associated with cell-cycle arrest (Fig. 7), these data supposed that multiple cellular factors inherent to cell-cycle progression and apoptosis were affected by aberration of *Tspo2*. Actually, the mRNA expression levels of the genes involved in negative control of erythropoiesis through apoptosis, such as *Fas*, *Fasl*, and *Trail* (2, 31, 32), were higher in *Tspo2*^−/−^ MEDEP cells compared with control cells through the maturation process with an exception at 24 h following induction (Fig. S4). mRNA levels of *p16* and *p53* involved in cell-cycle progression and apoptosis of erythroid cells (1, 33–35) were also higher in *Tspo2*^−/−^ cells (Fig. S4).

Integrating the findings from MEDEP cells, *Tspo2*^−/−^ mice, and HK dogs implies that the *Tspo2* defect causes the delays in multifaceted events during terminal erythroid differentiation and cell-cycle progression.

## Discussion

The findings from the present study revealed an important *in vivo* functional role for TSPO2 in both maturation and proliferation of late-stage erythroblasts. The noted defects are prominent in basophilic, polychromatic, and orthochromatic erythroblasts, the erythroid differentiation stages at which TSPO2 is highly expressed (19). Considering the reduced cell proliferation and hemoglobinization in *Tspo2*^−/−^ MEDEP cells and anemic phenotypes in *Tspo2*^−/−^ mice, the TSPO2 defect may reduce the efficiency of RBC production by ∼50%. The finding that key erythroid genes in terminal erythropoiesis, such as *Gata1*, *Klf1*, *TfR*, *Alas2*, *Hba*, *Hbb*, and *Setd8* (1–3, 36), are expressed at similar levels in control and *Tspo2*^−/−^ MEDEP cells implies that TSPO2-deficient erythroblasts are able to execute principal aspects of the terminal erythropoiesis program. As there is no reduction in the number of cell divisions prior to enucleation in TSPO2-deficient cells, perturbed maturation and cell proliferation due to the TSPO2 defects are most likely attributable to a delayed cell-cycle progression associated with impaired cytokinesis and death of the cells with cytokinesis failure. Although TSPO2 has originally been suggested to have a role in cholesterol redistribution in erythroblasts (19), the functional sequela of this observation was not defined. Our findings have implicated a major functional role for TSPO2 in erythropoiesis.

Because cholesterol is an essential component of cell membranes, proliferating cells have highly active cholesterol metabolism. Indeed, frequent hypocholesterolemia in patients with chronic anemias (24) and acute leukemia (37) suggests increased demand for cholesterol in proliferation of hematopoietic cells. In cytokinesis, cholesterol is essential for vesicular trafficking to form a membrane domain at the cleavage furrow ingression (38, 39) and mid-body tubules, a novel membrane-bound intracellular bridge (40). Lack of availability of cholesterol therefore causes cells to spend a prolonged period in cytokinesis and an increase in multinucleation (40). Furthermore, earlier studies had demonstrated that cholesterol starvation in human leukemia HL-60 cells induces the inhibition of cell proliferation and cell-cycle arrest selectively in G_2_/M phase and the consequent formation of multinucleated polyploid cells (41–43). The phenotypes observed in several distinct models in our study are compatible with such reported manifestations and confirm that cholesterol is essential for cell division and cell-cycle progression in hematopoiesis. Notably, a profound reduction in cholesterol content in *Tspo2*^−/−^ MEDEP cells strongly suggests that the *Tspo2* aberration causes cholesterol depletion in maturing erythroblasts. Taken together, our findings with both *in vivo* and *in vitro* models imply that restricted availability or depletion of cholesterol is most likely the primary cause for the impairments in cell-cycle progression and cytokinesis due to the TSPO2 defect. Lack of reduction in total plasma cholesterol levels found in our *Tspo2*^−/−^ mice is likely due to the modest anemic phenotype compared with patients with more severe anemia and markedly increased erythropoietic activity that result in reductions in hemoglobin and total cholesterol of ∼70 and 50%, respectively (24). Further studies in *Tspo2*^−/−^ mice and MEDEP cells under various cholesterol levels will be required to investigate the role of TPSO2 in physiological maturation and proliferation of late-stage erythroblasts.

Moreover, it is intriguing that ablation of RhoA GTPase has previously been shown to play a significant role in cytokinesis and abscission through cleavage furrow, impairs cytokinesis, and leads to cell-cycle arrest and cell death in murine fetal erythroblasts. Cytokinesis failure in RhoA-deficient erythroblasts at basophilic to orthochromatic stages manifested as polyploidy, maturation delay, cell-cycle arrest in G_2_/M phase, and increased cell death (35). These erythroblast phenotypes are very similar to those observed in our mouse or dog models of TSPO2 deficiency. The major difference was that the failure of definitive erythropoiesis in RhoA deficiency caused profound reduction in maturing erythroblasts and fetal death, whereas TSPO2 deficiency caused modest and compensated anemia. Defective cytokinesis in RhoA-deficient erythroblasts resulted in increased phosphorylation of p53 and transcriptional up-regulation of p21, leading to cell-cycle arrest and apoptotic cell death (35). Although it is unclear how TSPO2 defect leads to cytokinesis failure, increased mRNA levels of genes inherent to the cell cycle or apoptosis, such as *p53*, *p16*, *Fas/Fasl*, and *Trail*, in maturing *Tspo2*^−/−^ MEDEP cells suggest that the downstream cell-cycle arrest and cell death caused by the TSPO2 defect occurs through a mechanism similar to that observed in RhoA-deficient erythroblasts (35) and/or through increased activities of other apoptotic pathways in late-stage erythroblasts (31–34).

Our work also demonstrated that the expression of cTSPO2 in K562 cells causes cholesterol accumulation in the ER. In general, the ER receives cholesterol from the plasma membrane, other intracellular organelles including the endocytic recycling compartment and the *trans*-Golgi network, or via *de novo* synthesis and then recycles it back to the plasma membrane through several distinct pathways (44–47). Excess cholesterol is esterified and stored as CEs in lipid droplets and is released again after esterification hydrolysis when needed. Interestingly, both of these conversions occur in the ER (46, 48), and it is suggested that excess cholesterol is exported from the ER rather than being esterified (48). As the appearance of TSPO2 parallels down-regulation of *de novo* synthesis of cholesterol during erythroblast maturation (19), we suggest that TSPO2 participates in redistribution, including accumulation of free cholesterol in the ER, from internal or extracellular resources to efficiently provide cholesterol to the plasma membrane. Our finding of a reduction in CEs and a simultaneous increase in free cholesterol in K562 cells expressing WT cTSPO2 and the decreased cholesterol availability in *Tspo2*^−/−^ MEDEP cells are supportive of this thesis and suggest that lipid droplets can be the intracellular source of cholesterol. Moreover, this assumption is consistent with role for a the cholesterol-enriched membrane domain that has been implicated in vesicular trafficking and cytokinesis (38–40). It is also compatible with the requirement of cholesterol in enucleation that involves endocytic vesicular trafficking, Rac GTPase–dependent assembly of lipid rafts, and coalescence of lipid rafts between reticulocytes and pyrenocytes (23, 49). On the other hand, cholesterol levels in the ER have been shown to selectively regulate secretory protein trafficking through the COPII vesicular transport pathway (50–52). Accordingly, a possible regulation of cholesterol levels in the ER by TSPO2 may also affect the ER-to-Golgi transport and the plasma membrane expression of some membrane proteins during erythropoiesis.

Thus, the TSPO2 defects result in immature RBC phenotypes, as demonstrated in HK dog RBCs (13, 17, 22), presumably due to the delayed and incomplete maturation. The HK RBC trait in dogs is a recessive phenotype that depends on the total loss of Na,K-ATPase in erythrocytes. However, the TSPO2 defects appear to have dominant effects in erythropoiesis, as demonstrated by the cTSPO2 contents in dog RBC membranes as well as the erythroid phenotypes in the knockout mice, although they have modest RBC phenotypes (12, 13, 15). One inconsistency in our models is that TSPO2 defects result in a larger size of nascent reticulocytes both in dog BM-derived erythroblasts and in MEDEP cells, whereas there was no significant difference in the size of circulating RBCs in knockout mice. This discrepancy may be attributable to a further maturation of released reticulocytes by phagolysosome expulsion of organelles (6) and the plasma membrane remodeling (7–9). Indeed, HK erythrocytes possess no remnant organelles, and HK and LK reticulocytes exhibit similar changes in the membrane protein composition by proteolysis or exosome expulsion during maturation into erythrocytes (12, 13). Thus, TSPO2 is essential for survival and coordinated maturation and proliferation of erythroblasts, whereas it is dispensable in further maturation of the enucleate progeny.

Apart from the cholesterol-related function, TSPO2 has recently been reported to be involved in the membrane transport of a heme analogue protoporphyrin IX or ATP in human RBCs by forming a supramolecular complex with the voltage-dependent anion channel and adenine nucleotide transporter (22, 53). A mitochondrial paralogous protein, TSPO, also forms a complex with voltage-dependent anion channel and adenine nucleotide transporter to mediate various mitochondrial functions, including cholesterol and porphyrin transport, generation of reactive oxygen species, and cell proliferation (20, 21). Although we have shown that the *TSPO2* mutations affect the cTSPO2 contents in canine RBCs, it remains unknown whether it has some effect on the functions and characteristics of mature RBCs. Further studies need to define whether TSPO2 is involved in the formation of a similar protein complex and its relevant membrane transport function in the ER in erythroblasts.

In conclusion, the present study has identified a regulatory role of TSPO2 on cell-cycle progression and cytokinesis in erythropoiesis. Although the precise mechanism remains to be fully defined, TSPO2 appears to play an essential role in maintaining cholesterol availability, leading to harmonized maturation and proliferation of late erythroblasts.

## Experimental procedures

### Animals

The dogs used in this study included 15 Japanese mongrel dogs with HK or LK RBCs (mixed breed of Shiba; Fig. 1*A*), a purebred Shiba with HK RBCs, and eight beagles with LK RBCs. These dogs appear clinically healthy, although the HK RBCs had a shortened lifespan, increased mean corpuscular volume, and normal mean corpuscular hemoglobin compared with the LK cells (15, 17, 18). *Tspo2*^−/−^ mice were produced as described below. Dogs and mice were kept at the animal experimentation facility of the Graduate School of Veterinary Medicine, Hokkaido University. All experimental procedures were reviewed and approved by the Laboratory Animal Experimentation Committee, Graduate School of Veterinary Medicine, Hokkaido University with approval numbers 15-0017, 15-0019, and 15-0023.

### Antibodies

Rabbit anti-canine TSPO2 (cTSPO2) antibody was raised against the synthetic C-terminal pentadecapeptide of cTSPO2 (NH_2_-CPNHHQPLPMGEKRD-COOH) and was purified by affinity chromatography on the antigen peptide-coupled HiTrap NSH-activated resin (GE Healthcare). Other antibodies used are anti-canine Na,K-ATPase α-subunit (12), anti-human stomatin (13), phycoerythrin (PE)-conjugated anti-mouse CD71 (BD Pharmingen), PE-Cy7–conjugated anti-mouse CD44 and FITC-conjugated anti-mouse TER119 (both from Tonbo Biosciences, Tokyo, Japan), and allophycocyanin (APC)-conjugated anti-mouse CD45.2, Gr1, and CD11b (all from BioLegend). Secondary antibodies labeled with Alexa Fluor 488 or 568 were obtained from Molecular Probes.

### Genome-wide linkage analysis and mutation analysis

Whole-genome genotyping was performed with CanineSNP50 BeadChip (Illumina) on the dogs described above. Genomic DNA was prepared from peripheral blood using a QIAamp DNA blood minikit (Qiagen). The SNP genotyping data were analyzed for homozygosity mapping by allelic association analysis using PLINK software (54). All exons of the candidate genes in the critical region (chromosome 12, 9.4–10.7 Mb; Fig. 1*B*) were amplified by PCR (primers listed in Table S1) from genomic DNA obtained from the HK and LK dogs, and their nucleotide sequences were determined. After defining the mutations in *TSPO2*, dogs were genotyped by KpnI digestion or sequencing of appropriate PCR fragments for the C40Y or VFT mutations, respectively. DNA sequences of WT, C40Y, and VFT cTSPO2 were deposited in GenBank^TM^ with accession numbers MN397823, MN397824, and MN397825, respectively.

### Generation of K562 cells stably expressing cTSPO2

cTSPO2 and the mutant cTSPO2 cDNAs were amplified by PCR, cloned into a lentiviral vector, and transduced into K562 cells to produce cells stably expressing the WT, C40Y, and VFT cTSPO2 as described previously (55). We confirmed that the expression of endogenous human TSPO2 was at an undetectable level in control K562 cells by PCR as reported previously (19).

### Production of Tspo2 knockout mice

The *Tspo2* mutant mice were generated by CRISPR/Cas9-mediated gene knockout (56) by injecting the exon 3-specific sgRNA (5′-CTTTGTAGGTGTGCCC-3′), Cas9 mRNA, and the single-strand donor DNA (5′-CTTTGTAGGTGTGCCCtaatagactagtctagCTGcag-3′) into the pronucleus of embryos of C57BL/6 mice. The donor DNA contained several mutations to create terminating codons and restriction sites (shown in lowercase) at the targeted region. The founder mouse obtained was compound heterozygous for a deletion mutation of 105 nucleotides (Δ105, g.1374_1478del) and an insertion mutation of 2 nucleotides (Ins2, g.1486_1487insCG). This mouse was backcrossed to C57BL/6 mice to generate heterozygous (*Tspo2*^+/−^) offspring for two independent mutations Δ105 and Ins2, and mice were crossed within the same strain to obtain WT (*Tspo2*^+/+^), heterozygous (*Tspo2*^+/−^), and homozygous (*Tspo2*^−/−^) progeny. In this study, we used mice with the Δ105 mutation. Mice were genotyped by PCR followed by digestion of the PCR products with PstI (Fig. S2).

### Complete blood count

The complete blood count was carried out for blood from animals using the hematology analyzer ProCyte (IDEXX Laboratories). Reticulocytes were counted after supravital staining with new methylene blue.

### Morphology and culture of bone marrow cells

BM cells from dogs and mice were stained with Wright–Giemsa for morphological examination. Mononuclear cells from dog BM were cultured using two-phase liquid culture (57). Briefly, the cells were grown for 7 days in Iscove's modified Dulbecco's medium containing 15% fetal bovine serum, 10 μg/ml insulin (Sigma), 1 μm β-mercaptoethanol, 10 ng/ml stem cell factor, and 1 ng/ml interleukin-3 (both from R&D Systems), 200 μg/ml holo-transferrin (Wako Pure Chemicals, Japan), and 3 units/ml erythropoietin (Kyowa-Kirin, Japan) and then differentiated (second phase) in a similar medium supplemented with 10 units/ml erythropoietin, 1 μm triiodothyronine, and 800 μg/ml transferrin for 10 days. At appropriate intervals during the second phase of culture, cytospin smears of cells in culture were stained with Wright–Giemsa, and the size of extruded nuclei (pyrenocytes) and nascent reticulocytes generated by enucleating cells were determined using ImageJ (National Institutes of Health).

### Preparation of Tspo2^−/−^ MEDEP-BRC5 cell line and culture

MEDEP-BRC5 cells (25) were purchased from the Riken Bioresource Research Center (Tsukuba, Japan). *Tspo2*^−/−^ MEDEP-BRC5 cells were generated by the CRISPR/Cas9-mediated gene knockout technique (58). Briefly, mouse *Tspo2* exon 1–specific sgRNA (5′-GTCAGCATCCAGTCGGGTGTG-3′) was inserted into BbsI-treated pX330-U6-Chimeric_BB-Cbh-hSpCas9 (Addgene). MEDEP cells were transfected with this plasmid and pCDH-CMV-MCS-puro (Addgene). Two days later, cells were selected for 48 h with 1 μg/ml puromycin and cloned by limiting dilution. We obtained two independent clones of *Tspo2*^−/−^ cells. The clone KO13 was compound heterozygous for p.Arg29fs (c.85_88del) that resulted in a premature termination and p.Ser27X (c.80_90del) mutations. The other clone KO26 was homozygous for p.Cys30X (c.89_90del) mutation. The derived cells were maintained and induced into terminal differentiation as reported previously (25). Pyrenocyte and reticulocyte sizes were measured as described above, and hemoglobin contents were measured (59). Because clones KO13 and KO26 showed impairments similar to each other in cell proliferation and hemoglobinization, further studies were carried out on the clone KO13.

### Flow cytometry of bone marrow cells and cultured cells

Flow cytometric analysis for expression of phenotypic markers of erythropoiesis followed the established protocol (29, 30). Bone marrow cells from mice or cultured MEDEP cells (0.5 ∼1 × 10^6^) were stained with PE-conjugated anti-CD71, FITC-conjugated anti-TER119, and PE-Cy7–conjugated anti-CD44. After washing with PBS plus 0.2% BSA, cells were reacted with APC-conjugated streptavidin in PBS plus 0.2% BSA followed by washing in the same buffer. Bone marrow cells were collected from femurs of 20-week-old mice. The cells were washed in PBS plus 0.2% BSA and stained with the same antibodies described above and APC-conjugated anti-CD45.2/Gr1/CD11b antibodies followed by washing in PBS plus 0.2% BSA. Finally, these cells were stained with the viability marker propidium iodide (PI) and analyzed on a FACSVerse flow cytometer (BD Biosciences). Data were analyzed by FlowJo version 10 (FlowJo).

### Cholesterol uptake and intracellular distribution

The uptake of NBD-cholesterol was carried out as described previously (19). Intracellular unesterified cholesterol and CEs were stained with filipin III and Nile Red, respectively. Endocytosis of FITC-conjugated transferrin was also examined (60). Cholesterol accumulation in the ER of K562 cells was analyzed by SREBP-mediated stimulation of the reporter gene driven by the LDLR promoter (28, 44). A reporter plasmid pGL4LDLR-Luc was constructed as reported previously (28). Briefly, −308 to −61 of human LDLR promoter region was amplified by PCR from genomic DNA of K562 cells, cloned, and inserted into pGL4.17 vector (Promega). K562 cells stably expressing WT, C40Y, or VFT cTSPO2 and the cells transduced with empty vector were transfected with pGL4LDLR-Luc. Simultaneously, an internal control luciferase plasmid pGL4.74 (Promega) was also transfected for normalization. Luciferase activity was determined using a Dual-Luciferase reporter assay system (Promega).

### Analyses of cell division, cell cycle, and apoptotic cell death

Flow cytometric analysis for cell division using CellTrace Violet (Thermo Fisher) was carried out as recommended by the manufacturer using a FACSAriaII cell sorter (BD Bioscience), and the number of cell divisions was estimated as reported previously (10). For cell-cycle analysis, cells were harvested and washed in PBS followed by fixation in chilled 70% ethanol plus PBS. Fixed cells were incubated at 37 °C for 30 min in PBS containing 250 μg/ml RNase. Then PI was added to obtain 50 μg/ml, and the cells were incubated for 30 min on ice followed by flow cytometric analysis. Apoptotic cell death was analyzed by flow cytometry using the Annexin V-FITC apoptosis detection kit (BioVision).

### Other procedures

Immunofluorescence microscopy, SDS-PAGE and immunoblotting, PCR, quantitative RT-PCR, transfection of the plasmids into cultured cells, protein concentration determination, and total cholesterol measurement were performed as described previously (13, 55, 61).

### Statistical analysis

Unpaired two-tailed statistical analysis or Mann–Whitney *U* test for nonparametric data was performed on GraphPad Prism. All analyses were considered statistically significant at *p* < 0.05.

## Data availability

DNA sequences were deposited in GenBank^TM^ with accession numbers MN397823, MN397824, and MN397825. All other data are contained within the article and supporting information.

## Supplementary Material

Supporting Information

## References

[1] Dzierzak E., and Philipsen S. (2013) Erythropoiesis: development and differentiation. Cold Spring Harb. Perspect. Med. 3, a011601 10.1101/cshperspect.a011601 23545573 PMC3684002

[2] Papayannopoulou T., and Migliaccio A. R. (2017) Biology of erythropoiesis, erythroid differentiation, and maturation. in Hematology: Basic Principles and Practice, 7th Ed. (Hoffman R., Benz E. J. Jr., Silberstein. L. E., Heslop H. E., Weitz J. I., Anastasi J., Salama M. E., and Abutalib S. A., eds) Elsevier, Philadelphia, PA

[3] Malik J., Lillis J. A., Couch T., Getman M., and Steiner L. A. (2017) The methyltransferase Setd8 is essential for erythroblast survival and maturation. Cell Rep. 21, 2376–2383 10.1016/j.celrep.2017.11.011 29186677 PMC7748363

[4] Gnanapragasam M. N., McGrath K. E., Catherman S., Xue L., Palis J., and Bieker J. J. (2016) EKLF/KLF1-regulated cell cycle exit is essential for erythroblast enucleation. Blood 128, 1631–1641 10.1182/blood-2016-03-706671 27480112 PMC5034741

[5] Zhao B., Mei Y., Schipma M. J., Roth E. W., Bleher R., Rappoport J. Z., Wickrema A., Yang J., and Ji P. (2016) Nuclear condensation during mouse erythropoiesis requires caspase-3-mediated nuclear opening. Dev. Cell 36, 498–510 10.1016/j.devcel.2016.02.001 26954545 PMC4785602

[6] Holm T. M., Braun A., Trigatti B. L., Brugnara C., Sakamoto M., Krieger M., and Andrews N. C. (2002) Failure of red blood cell maturation in mice with defects in the high-density lipoprotein receptor SR-BI. Blood 99, 1817–1824 10.1182/blood.V99.5.1817 11861300

[7] Lazarides E., and Moon R. T. (1984) Assembly and topogenesis of the spectrin-based membrane skeleton in erythroid development. Cell 37, 354–356 10.1016/0092-8674(84)90364-7 6233006

[8] Hanspal M., and Palek J. (1987) Synthesis and assembly of membrane skeletal proteins in mammalian red cell precursors. J. Cell Biol. 105, 1417–1424 10.1083/jcb.105.3.1417 3654760 PMC2114789

[9] Mohandas N., and Gallagher P. G. (2008) Red cell membrane: past, present, and future. Blood 112, 3939–3948 10.1182/blood-2008-07-161166 18988878 PMC2582001

[10] Sankaran V. G., Ludwig L. S., Sicinska E., Xu J., Bauer D. E., Eng J. C., Patterson H. C., Metcalf R. A., Natkunam Y., Orkin S. H., Sicinski P., Lander E. S., and Lodish H. F. (2012) Cyclin D3 coordinates the cell cycle during differentiation to regulate erythrocyte size and number. Genes Dev. 26, 2075–2087 10.1101/gad.197020.112 22929040 PMC3444733

[11] Chan P. C., Calabrese V., and Theil L. S. (1964) Species differences in the effect of sodium and potassium ions on the ATPase of erythrocyte membranes. Biochim. Biophys. Acta 79, 424–426 10.1016/0926-6577(64)90028-2 14163532

[12] Inaba M., and Maede Y. (1986) Na,K-ATPase in dog red cells: immunological identification and maturation-associated degradation by the proteolytic system. J. Biol. Chem. 261, 16099–16105 3023340

[13] Komatsu T., Sato K., Otsuka Y., Arashiki N., Tanaka K., Tamahara S., Ono K., and Inaba M. (2010) Parallel reductions in stomatin and Na,K-ATPase through the exosomal pathway during reticulocyte maturation in dogs: stomatin as a genotypic and phenotypic marker of high K^+^ and low K^+^ red cells. J. Vet. Med. Sci. 72, 893–901 10.1292/jvms.10-0030 20215716

[14] Inaba M., and Messick J. B. (2010) Red blood cell membrane defects. in Schalm's Veterinary Hematology, 6th Ed. (Weiss D. J., and Wardrop K. J., eds) pp. 187–195, Wiley-Blackwell, Hoboken, NJ

[15] Maede Y., Inaba M., and Taniguchi N. (1983) Increase of Na-K-ATPase activity, glutamate, and aspartate uptake in dog erythrocytes associated with hereditary high accumulation of GSH, glutamate, glutamine, and aspartate. Blood 61, 493–499 10.1182/blood.V61.3.493.493 6297638

[16] Maede Y., and Inaba M. (1985) (Na,K)-ATPase and ouabain binding in reticulocytes from dogs with high K and low K erythrocytes and their changes during maturation. J. Biol. Chem. 260, 3337–3343 2982856

[17] Maede Y., and Inaba M. (1987) Energy metabolism in canine erythrocytes associated with inherited high Na^+^- and K^+^-stimulated adenosine triphosphatase activity. Am. J. Vet. Res. 48, 114–118 3030164

[18] Inaba M., and Maede Y. (1989) Inherited persistence of immature type pyruvate kinase and hexokinase isozymes in dog erythrocytes. Comp. Biochem. Physiol. B. 92, 151–156 10.1016/0305-0491(89)90328-3 2706933

[19] Fan J., Rone M. B., and Papadopoulos V. (2009) Translocator protein 2 is involved in cholesterol redistribution during erythropoiesis. J. Biol. Chem. 284, 30484–30497 10.1074/jbc.M109.029876 19729679 PMC2781603

[20] Papadopoulos V., Baraldi M., Guilarte T. R., Knudsen T. B., Lacapère J.-J., Lindemann P., Norenberg M. D., Nutt D., Weizman A., Zhang M.-R., and Gavish M. (2006) Translocator protein (18 kDa): new nomenclature for the peripheral-type benzodiazepine receptor based on its structure and molecular function. Trends Pharmacol. Sci. 27, 402–409 10.1016/j.tips.2006.06.005 16822554

[21] Rupprecht R., Papadopoulos V., Rammes G., Baghai T. C., Fan J., Akula N., Groyer G., Adams D., and Schumacher M. (2010) Translocator protein (18 kDa) (TSPO) as a therapeutic target for neurological and psychiatric disorders. Nat. Rev. Drug Discov. 9, 971–988 10.1038/nrd3295 21119734

[22] Marginedas-Freixa I., Hattab C., Bouyer G., Halle F., Chene A., Lefevre S. D., Cambot M., Cueff A., Schmitt M., Gamain B., Lacapere J. J., Egee S., Bihel F., Le Van Kim C., and Ostuni M. A. (2016) TSPO ligands stimulate ZnPPIX transport and ROS accumulation leading to the inhibition of *P. falciparum* growth in human blood. Sci. Rep. 6, 33516 10.1038/srep33516 27641616 PMC5027585

[23] Konstantinidis D. G., Pushkaran S., Johnson J. F., Cancelas J. A., Manganaris S., Harris C. E., Williams D. A., Zheng Y., and Kalfa T. A. (2012) Signaling and cytoskeletal requirements in erythroblast enucleation. Blood 119, 6118–6127 10.1182/blood-2011-09-379263 22461493 PMC3383020

[24] Shalev H., Kapelushnik J., Moser A., Knobler H., and Tamary H. (2007) Hypocholesterolemia in chronic anemias with increased erythropoietic activity. Am. J. Hematol. 82, 199–202 10.1002/ajh.20804 17039515

[25] Hiroyama T., Miharada K., Sudo K., Danjo I., Aoki N., and Nakamura Y. (2008) Establishment of mouse embryonic stem cell-derived erythroid progenitor cell lines able to produce functional red blood cells. PLoS ONE 3, e1544 10.1371/journal.pone.0001544 18253492 PMC2212133

[26] Gautier E.-F., Leduc M., Ladli M., Schultz V. P., Lefèvre C., Boussaid I., Fontenay M., Lacombe C., Verdier F., Guillonneau F., Hillyer C. D., Mohandas N., Gallagher P. G., and Mayeux P. (2020) Comprehensive proteomic analysis of murine terminal erythroid differentiation. Blood Adv. 4, 1464–1477 10.1182/bloodadvances.2020001652 32282884 PMC7160260

[27] Robinson M. S. (2015) Forty years of clathrin-coated vesicles. Traffic 16, 1210–1238 10.1111/tra.12335 26403691

[28] Shimano H., Horton J. D., Shimomura I., Hammer R. E., Brown M. S., and Goldstein J. L. (1997) Isoform 1c of sterol regulatory element binding protein is less active than isoform 1a in livers of transgenic mice and in cultured cells. J. Clin. Invest. 99, 846–854 10.1172/JCI119248 9062341 PMC507891

[29] Chen K., Liu J., Heck S., Chasis J. A., An X., and Mohandas N. (2009) Resolving the distinct stages in erythroid differentiation based on dynamic changes in membrane protein expression during erythropoiesis. Proc. Natl. Acad. Sci. U.S.A. 106, 17413–17418 10.1073/pnas.0909296106 19805084 PMC2762680

[30] Liu J., Zhang J., Ginzburg Y., Li H., Xue F., De Franceschi L., Chasis J. A., Mohandas N., and An X. (2013) Quantitative analysis of murine terminal erythroid differentiation *in vivo*: novel method to study normal and disordered erythropoiesis. Blood 121, e43 10.1182/blood-2012-09-456079 23287863 PMC3578961

[31] De Maria R., Testa U., Luchetti L., Zeuner A., Stassi G., Pelosi E., Riccioni R., Felli N., Samoggia P., and Peschle C. (1999) Apoptotic role of Fas/Fas ligand system in the regulation of erythropoiesis. Blood 93, 796–803 10.1182/blood.V93.3.796 9920828

[32] Zamai L., Secchiero P., Pierpaoli S., Bassini A., Papa S., Alnemri E. S., Guidotti L., Vitale M., and Zauli G. (2000) TNF-related apoptosis-inducing ligand (TRAIL) as a negative regulator of normal human erythropoiesis. Blood 95, 3716–3724 10845902

[33] Minami R., Muta K., Umemura T., Motomura S., Abe Y., Nishimura J., and Nawata H. (2003) p16^INK4a^ induces differentiation and apoptosis in erythroid lineage cells. Exp. Hematol. 31, 355–362 10.1016/S0301-472X(03)00040-7 12763133

[34] Peller S., Frenkel J., Lapidot T., Kahn J., Rahimi-Levene N., Yona R., Nissim L., Goldfinger N., Sherman D. J., and Rotter V. (2003) The onset of p53-dependent apoptosis plays a role in terminal differentiation of human normoblasts. Oncogene 22, 4648–4655 10.1038/sj.onc.1206541 12879009

[35] Konstantinidis D. G., Giger K. M., Risinger M., Pushkaran S., Zhou P., Dexheimer P., Yerneni S., Andreassen P., Klingmüller U., Palis J., Zheng Y., and Kalfa T. A. (2015) Cytokinesis failure in RhoA-deficient mouse erythroblasts involves actomyosin and midbody dysregulation and triggers p53 activation. Blood 126, 1473–1482 10.1182/blood-2014-12-616169 26228485 PMC4573870

[36] Malik J., Getman M., and Steiner L. A. (2015) Histone methyltransferase Setd8 represses Gata2 expression and regulates erythroid maturation. Mol. Cell. Biol. 35, 2059–2072 10.1128/MCB.01413-14 25848090 PMC4438238

[37] Vitols S., Gahrton G., Björkholm M., and Peterson C. (1985) Hypocholesterolaemia in malignancy due to elevated low-density-lipoprotein-receptor activity in tumour cells: evidence from studies in patients with leukaemia. Lancet 2, 1150–1154 10.1016/s0140-6736(85)92679-0 2865616

[38] Ng M. M., Chang F., and Burgess D. R. (2005) Movement of membrane domains and requirement of membrane signaling molecules for cytokinesis. Dev. Cell 9, 781–790 10.1016/j.devcel.2005.11.002 16326390

[39] Montagnac G., Echard A., and Chavrier P. (2008) Endocytic traffic in animal cell cytokinesis. Curr. Opin. Cell Biol. 20, 454–461 10.1016/j.ceb.2008.03.011 18472411

[40] Kettle E., Page S. L., Morgan G. P., Malladi C. S., Wong C. L., Boadle R. A., Marsh B. J., Robinson P. J., and Chircop M. (2015) A cholesterol-dependent endocytic mechanism generates midbody tubules during cytokinesis. Traffic 16, 1174–1192 10.1111/tra.12328 26399547

[41] Martínez-Botas J., Suárez Y., Ferruelo A. J., Gómez-Coronado D., and Lasunción M. A. (1999) Cholesterol starvation decreases p34^cdc2^ kinase activity and arrests the cell cycle at G_2_. FASEB J. 13, 1359–1370 10.1096/fasebj.13.11.1359 10428760

[42] Fernández C., Lobo M. V., Gómez-Coronado D., and Lasunción M. A. (2004) Cholesterol is essential for mitosis progression and its deficiency induces polyploid cell formation. Exp. Cell Res. 300, 109–120 10.1016/j.yexcr.2004.06.029 15383319

[43] Fernández C., Martin M., Gómez-Coronado D., and Lasunción M. A. (2005) Effects of distal cholesterol biosynthesis inhibitors on cell proliferation and cell cycle progression. J. Lipid Res. 46, 920–929 10.1194/jlr.M400407-JLR200 15687348

[44] Ikonen E. (2008) Cellular cholesterol trafficking and compartmentalization. Nat. Rev. Mol. Cell Biol. 9, 125–138 10.1038/nrm2336 18216769

[45] Mukherjee S., Zha X., Tabas I., and Maxfield F. R. (1998) Cholesterol distribution in living cells: fluorescence imaging using dehydroergosterol as a fluorescent cholesterol analog. Biophys. J. 75, 1915–1925 10.1016/S0006-3495(98)77632-5 9746532 PMC1299862

[46] Maxfield F. R., and Tabas I. (2005) Role of cholesterol and lipid organization in disease. Nature 438, 612–621 10.1038/nature04399 16319881

[47] Rone M. B., Fan J., and Papadopoulos V. (2009) Cholesterol transport in steroid biosynthesis: role of protein-protein interactions and implications in disease states. Biochim. Biophys. Acta 1791, 646–658 10.1016/j.bbalip.2009.03.001 19286473 PMC2757135

[48] Zhang S., Glukhova S. A., Caldwell K. A., and Caldwell G. A. (2017) NCEH-1 modulates cholesterol metabolism and protects against α-synuclein toxicity in a *C. elegans* model of Parkinson's disease. Hum. Mol. Genet. 26, 3823–3836 10.1093/hmg/ddx269 28934392 PMC5886139

[49] Keerthivasan G., Small S., Liu H., Wickrema A, Crispino J. D. (2010) Vesicle trafficking plays a novel role in erythroblast enucleation. Blood 116, 3331–3340 10.1182/blood-2010-03-277426 20644112 PMC2995360

[50] Espenshade P. J., Li W. P., and Yabe D. (2002) Sterol block binding of COPII proteins to SCAP, thereby controlling SCAP sorting in ER. Proc. Natl. Acad. Sci. U.S.A. 99, 11694–11699 10.1073/pnas.182412799 12193656 PMC129331

[51] Runz H., Miura K., Weiss M., and Pepperkok R. (2006) Sterols regulate ER-export dynamics of secretory cargo protein ts-O45-G. EMBO J. 25, 2953–2965 10.1038/sj.emboj.7601205 16794576 PMC1500972

[52] Bonnon C., Wendeler M. W., Paccaud J.-P., and Hauri H.-P. (2010) Selective export of human GPI-anchored proteins from the endoplasmic reticulum. J. Cell Sci. 123, 1705–1715 10.1242/jcs.062950 20427317

[53] Marginedas-Freixa I., Alvarez C. L., Moras M., Leal Denis M. F., Hattab C., Halle F., Bihel F., Mouro-Chanteloup I., Lefevre S. D., Le Van Kim C., Schwarzbaum P. J., and Ostuni M. A. (2018) Human erythrocytes release ATP by a novel pathway involving VDAC oligomerization independent of pannexin-1. Sci. Rep. 8, 11384 10.1038/s41598-018-29885-7 30061676 PMC6065367

[54] Purcell S., Neale B., Todd-Brown K., Thomas L., Ferreira M. A. R., Bender D., Maller J., Sklar P., de Bakker P. I. W., Daly M. J., and Sham P. C. (2007) PLINK: a tool set for whole-genome association and population-based linkage analyses. Am. J. Hum. Genet. 81, 559–575 10.1086/519795 17701901 PMC1950838

[55] Ito D., Koshino I., Arashiki N., Adachi H., Tomihari M., Tamahara S., Kurogi K., Amano T., Ono K., and Inaba M. (2006) Ubiquitylation-independent ER-associated degradation of an AE1 mutant associated with dominant hereditary spherocytosis in cattle. J. Cell Sci. 119, 3602–3612 10.1242/jcs.03101 16912075

[56] Yang H., Wang H., and Jaenisch R. (2014) Generating genetically modified mice using CRISPR/Cas-mediated genome engineering. Nat. Protoc. 9, 1956–1968 10.1038/nprot.2014.134 25058643

[57] Ohene-Abuakwa Y., Orfali K. A., Marius C., and Ball S. E. (2005) Two-phase culture in Diamond Blackfan anemia: localization of erythroid defect. Blood 105, 838–846 10.1182/blood-2004-03-1016 15238419

[58] Cong L., Ran F. A., Cox D., Lin S., Barretto R., Habib N., Hsu P. D., Wu X., Jiang W., Marraffini L. A., and Zhang F. (2013) Multiplex genome engineering using CRISPR/Cas systems. Science 339, 819–823 10.1126/science.1231143 23287718 PMC3795411

[59] Cioe L., McNab A., Hubbell H. R., Meo P., Curtis P., and Rovera G. (1981) Differential expression of the globin genes in human leukemia K562(S) cells induced to differentiate by hemin or butyric acid. Cancer Res. 41, 237–243 6934848

[60] Wang C. C., Sato K., Otsuka Y., Otsu W., and Inaba M. (2012) Clathrin-mediated endocytosis of mammalian erythroid AE1 anion exchanger facilitated by a YXXΦ or a noncanonical YXXXΦ motif in the N-terminal stretch. J. Vet. Med. Sci. 74, 17–25 10.1292/jvms.11-0345 21873807

[61] Otsu W., Kurooka T., Otsuka Y., Sato K., and Inaba M. (2013) A new class of endoplasmic reticulum export signal Φ*X*Φ*X*Φ for transmembrane proteins and its selective interaction with Sec24C. J. Biol. Chem. 288, 18521–18532 10.1074/jbc.M112.443325 23658022 PMC3689993

