# Supplementary material for: Cholesterol-binding protein TSPO2 coordinates maturation and proliferation of terminally differentiating erythroblasts
Source: J Biol Chem. 2020 May 1;295(23):8048–63. doi: 10.1074/jbc.RA119.011679 (PMC7278357; doi:10.1074/jbc.RA119.011679)
Supplement: Supporting Information [file supp_295_23_8048__index.html]

Cholesterol-binding protein TSPO2 coordinates maturation and proliferation of terminally differentiating erythroblasts — TSPO2 is essential for terminal erythroid differentiation — Supporting Information 

# Cholesterol-binding protein TSPO2 coordinates maturation and proliferation of terminally differentiating erythroblasts

## Supporting Information

- Supporting Information (to be published online) - Table S1 and Figs. S1-S4
