# Supplementary material for: Cholesterol-binding protein TSPO2 coordinates maturation and proliferation of terminally differentiating erythroblasts
Source: J Biol Chem. 2020 May 1;295(23):8048–63. doi: 10.1074/jbc.RA119.011679 (PMC7278357; doi:10.1074/jbc.RA119.011679)
Supplement: Supporting Information [file supp_RA119.011679_156805_2_supp_514822_q94zwc.pdf]

Supporting Information

Cholesterol-binding protein TSPO2 coordinates maturation and proliferation of terminally differentiating erythroblasts

Benjaporn Kiatpakdee[1], Kota Sato[1], Yayoi Otsuka[1], Nobuto Arashiki[1], Yuqi Chen[1], Takuya Tsumita[1], Wataru Otsu[1], Akito Yamamoto[1], Reo Kawata[1], Jumpei Yamazaki[1], Yoshikazu Sugimoto[2], Kensuke Takada[1], Narla Mohandas[3], Mutsumi Inaba[1]*

From the [1]Laboratory of Molecular Medicine, Graduate School of Veterinary Medicine, Hokkaido University, Sapporo, 060-0818, Japan; [2]Shirakawa Institute of Animal Genetics, Shirakawa, 961-8061, Japan; [3]Red Cell Physiology Laboratory, New York Blood Center, New York, NY 10065.

*Corresponding author

Table S1. Oligonucleotide primers used in this study

Genes	GenBank Accession numbers	Primer ID	Nucleotide sequences	Purposes
Canine *UNC5CL*	NC_006594.3	UNC5CLex1F	F: 5'-CAAGATCCTTGGCCTTGGCTGGC-3'	Genomic DNA sequence analysis
		UNC5CLex1R	R: 5'-GTCCAACACACAGTGAACAAC-3'	Genomic DNA sequence analysis
		UNC5CLex2F	F: 5-TAGGGGTGCACCTGCCTGCCAG-3'	Genomic DNA sequence analysis
		UNC5CLex2R	R: 5'-CCATACAGAAAGGCGAAGATGAG-3'	Genomic DNA sequence analysis
		UNC5CLex3F	F: 5'-GGTCCCAGGGTCCCCTGTATCAG-3'	Genomic DNA sequence analysis
		UNC5CLex3R	R: 5'-CCCTCTCCTAGGGCTCCTTGCTGC-3'	Genomic DNA sequence analysis
		UNC5CLex45F	F: 5'-TGTGGAAGACCCCGCTTCTGAG-3'	Genomic DNA sequence analysis
		UNC5CLex45R	R: 5'-CCTITGCCCAGGGCTCTCCCAGG-3'	Genomic DNA sequence analysis
		UNC5CLex67F	F: 5'-CTCAGGGGATGTCTCATGATCC-3'	Genomic DNA sequence analysis
		UNC5CLex67R	R: 5'-ACCCCCACCCTCACGCCAGTACC-3'	Genomic DNA sequence analysis
		UNC5CLex8F	F: 5'-GGAGGGGGTGGATCTGGCCCCAG-3'	Genomic DNA sequence analysis
		UNC5CLex8R	R: 5'-CATGCCCTGAGCGCTGCGGACAGC-3'	Genomic DNA sequence analysis
		UNC5CLex8F3	F: 5'-ACCTCGGGTCTCAAGGTTC-3'	Genomic DNA sequence analysis
	XM_538907.2	5UTRcUNC5CL	F: 5'-TGGCAGCACGAGTGTCCAGGCGG-3'	cDNA cloning
		3UTRcUNC5CL	R: 5'-ACAGCATCCCCACCCCAGCCCAC-3'	cDNA cloning
Canine *TSPO2*		cTSPO2ex1r	R: 5'-CATACCAGGTAGGAGGCATAGCCC-3'	Genomic DNA sequence analysis
		cTSPO2int1f	F: 5'-TCTGGCCTCCTCAGGGATGG-3'	Genomic DNA sequence analysis
		cTSPO2int2r	R: 5'-CCTGGTGAGCCAAGTGCCTG-3'	Genomic DNA sequence analysis
		5UTRcTSPO2	F: 5'-AGATTTTGCCTCTGTGAGGGGA-3'	cDNA cloning
		3UTRcTSPO2	R: 5'-GCCATCCTCCCTCCTCTCCTGTCC-3'	cDNA cloning
Canine *APOBEC2*	NC_006594.3	APOBEC2ex1F	F: 5'-GCCCTAGGAACTCGCTGCCTCTC-3'	Genomic DNA sequence analysis
		APOBEC2ex1R	R: 5'-CCCCCAACCCACGTTCTTCTACG-3'	Genomic DNA sequence analysis
		APOBEC2ex2F	F: 5'-GCTCATCATTCTCTCTTCTTCCCTG-3'	Genomic DNA sequence analysis
		APOBEC2ex2R	R: 5'-CAGCCCTCCACCCAAGCTAAGGGC-3'	Genomic DNA sequence analysis
	XM 538909.3	5UTRCAPOBEC2	F: 5'-TGACTGCAGAGCCATCACCCCTCC-3'	cDNA cloning
		3UTRCAPOBEC2	R: 5'-CTGGTGGCAGCAGGCAGACGTGAG-3'	cDNA cloning
Canine *C12H6Orf130*	NC_006594.3	C12H6orf130ex12F	F: 5'-CCTTCCTTTTCCCTTCTCAGCTGGAG-3'	Genomic DNA sequence analysis
		C12H6orf130ex12R	R: 5-CAGGAGGCCATGATGCTGCTCTAC-3'	Genomic DNA sequence analysis
		C12H6orf130ex34F	F: 5'-GATCGAAGAATGCAAATCGGTGC-3'	Genomic DNA sequence analysis
		C12H6orf130ex34R	R: 5'-TCAAGTCAGACAGCAAATCCCACC-3'	Genomic DNA sequence analysis
	XM 003431722.1	5UTRcC12H6orf130	F: 5'-AAGTTCTGGGAATAGCGACTCCG-3'	cDNA cloning
		3UTRcC12H6orf130	R: 5'-GGACACATCCACAGTGTGCATCTG-3'	cDNA cloning
Canine *NFYA*	NC_006594.3	NFYAex1F	F: 5'-GTCTTCTAGGATCTCCTGAGTG-3'	Genomic DNA sequence analysis
		NFYAex1R	R: 5'-CCATGTAGGTGGTGCCATTCTG-3'	Genomic DNA sequence analysis
		NFYAex2F	F: 5'-CATGTCTTGAACTATACATTTCC-3'	Genomic DNA sequence analysis

Genes	GenBank Accession numbers	Primer ID	Nucleotide sequences	Purposes
		NFYAex2R	R: 5'- AGATCCTACCACAAATAGCAC - 3'	Genomic DNA sequence analysis
		NFYAex3F	F: 5'-GAAGGCAGCAGAATAGTTCCAGTG-3'	Genomic DNA sequence analysis
		NFYAex3R	R: 5'-CTTCTTTCAGACATCTATTCTG-3'	Genomic DNA sequence analysis
		NFYAex45F	F: 5-CGAAAGAAACCAGTGCCAGCAGG-3	Genomic DNA sequence analysis
		NFYAex45R	R: 5-GCTCCAAGGATAAAAGAGGTGCC-3	Genomic DNA sequence analysis
		NFYAex6F	F: 5-GAAGAGGGACTAACTGTGTTCC-3	Genomic DNA sequence analysis
		NFYAex6R	R: 5-ATATACAGAAAGATAAATGACAG-3	Genomic DNA sequence analysis
		NFYAex7F	F: 5-GGCCAAGAAATAGTTTCCTTTGTC-3'	Genomic DNA sequence analysis
		NFYAex7R	R: 5-GTAATTTCAAAAGTACGTCCCTGCCC-3	Genomic DNA sequence analysis
		NFYAex8F	F: 5-GATAACCATGCTGGTTCCAACTAG-3	Genomic DNA sequence analysis
		NFYAex8R	R: 5-GTAAGAAGAGGACTCTGAATGCTC-3'	Genomic DNA sequence analysis
		NFYAex9F	F: 5-GGAGGAGTTAGAGCCTTGAGTTCC-3	Genomic DNA sequence analysis
		NFYAex9R	R: 5-CTGGAACAGTGGGAAGCAGTGGCG-3	Genomic DNA sequence analysis
	XM_003431723.1	5UTRcNFYA	F: 5'-ACAGGATTCTAACTTGGAGGGATC-3	cDNA cloning
		3UTRcNFYA	R: 5'-TGATCTGCTCCATCACATGGCCTG-3'	cDNA cloning
Canine *ADCY10L*	NC_006594.3	ADCY10Lex12F	F: 5'-CAACCCCGCCGCCGCCATGGCGAC-3'	Genomic DNA sequence analysis
		ADCY10Lex12R	R: 5'-CCTGTTGTTACAGACGCTCGCTC-3'	Genomic DNA sequence analysis
	XM_846097.2	5UTRCADCY10L	F: 5'-GGCAGCGCCGCCTCTGTGGCCC-3'	cDNA cloning
		3UTRCADCY10L	R: 5'-CGCCTCCTGGCTTCCCTACAGTG-3'	cDNA cloning
Canine *ADCY10*		ADCY10ex12F	F: 5'-GTATATGGCCAACTCTTGAGAATG-3'	Genomic DNA sequence analysis
		ADCY10ex12R	R: 5'-CCAGGTTGTCATTTATATTTTG-3'	Genomic DNA sequence analysis
		ADCY10ex12F3	F: 5'-GTAAATCCAGATACCTGTCATCC-3'	Genomic DNA sequence analysis
		ADCY10ex345F	F: 5'-CCAGACCCCACCCTTTGCATAG-3'	Genomic DNA sequence analysis
		ADCY10ex345R	R: 5'-CACCTGTCAACATAAACATACACAC-3'	Genomic DNA sequence analysis
		ADCY10ex345F3	F: 5'-GGTAAATGGCATCCTCAACC-3'	Genomic DNA sequence analysis
		ADCY10ex67F	F: 5'-GGCTAAACATGATATTGAATGTTG-3'	Genomic DNA sequence analysis
		ADCY10ex67R	R: 5'-GCCATCTACCCTGCCTGAAGAGC-3'	Genomic DNA sequence analysis
		ADCY10ex8F	F: 5'-GTGCCTCACTTTGACACGTCTGAC-3'	Genomic DNA sequence analysis
		ADCY10ex8R	R: 5'-GAACCATCTCTTCCACTTTCACC-3'	Genomic DNA sequence analysis
		ADCY10ex910F	F: 5'-ATCTTTGCAAAATAAACAGAGTGTC-3'	Genomic DNA sequence analysis
		ADCY10ex910R	R: 5'-CAATACTGGGCCCTACTATCCACC-3'	Genomic DNA sequence analysis
		ADCY10ex11F	F: 5'-GATGACTTCATTTCTAATCAGAAG-3'	Genomic DNA sequence analysis
		ADCY10ex11R	R: 5'-CTTATATTATGGCACTGGCTCTGC-3'	Genomic DNA sequence analysis
		ADCY10ex1213F	F: 5'-GTGGGCATCTTAGTCAGCAATCC-3'	Genomic DNA sequence analysis
		ADCY10ex1213R	R: 5'-CCAAATTACTAAGTGCAGTGACATCTG-3'	Genomic DNA sequence analysis
		ADCY10ex14F	F: 5'-GAATCGATTATCAACACAATGTATTC-3'	Genomic DNA sequence analysis

Genes	GenBank Accession numbers	Primer ID	Nucleotide sequences	Purposes
		ADCY10ex14R	R: 5'-CCTACCCAAGGTTCTCTTCTCTG-3'	Genomic DNA sequence analysis
		ADCY10ex15F	F: 5'-GTTTGCTACTGATAGCATAAAAGGCC-3'	Genomic DNA sequence analysis
		ADCY10ex15R	R: 5'-CACTTCTCTTTATTCATGTACCTGGAG-3'	Genomic DNA sequence analysis
		ADCY10ex16F	F: 5'-GAGGAGAAGTAGTCAAGAATTGG-3'	Genomic DNA sequence analysis
		ADCY10ex16R	R: 5'-CTGGTCTGCAGGTGAGGCAAGTGG-3'	Genomic DNA sequence analysis
Canine *ADCY10*		ADCY10ex1718F	F: 5'-GGTAATATGCCGAGAACAGTGGCTGC-3'	Genomic DNA sequence analysis
		ADCY10ex1718R	R: 5'-GGAATAATGGGGTAACCCTCAAG-3'	Genomic DNA sequence analysis
		ADCY10ex1718F3	F: 5'-CGCACTACTAGGGTCATCTGAATC-3'	Genomic DNA sequence analysis
		ADCY10ex1920F	F: 5'-CAGTGTATTATGTCCAGAGCTTACAGAG-3'	Genomic DNA sequence analysis
		ADCY10ex1920R	R: 5'-GGAATCTCTCTCCCTGCGTCTCAGG-3'	Genomic DNA sequence analysis
		ADCY10ex1920F3	F: 5'-GTCCAGAGCTTACAGAGTTTACA-3'	Genomic DNA sequence analysis
		ADCY10ex2122F	F: 5'-GTGTAGCAGGAATGTACAGTAGG-3'	Genomic DNA sequence analysis
		ADCY10ex2122R	R: 5'-CCCGAAGGATTCAGACCTCAGCGC-3'	Genomic DNA sequence analysis
		ADCY10ex2122F3	F: 5'-GAATGTACAGTAGGAACATTTTGC-3'	Genomic DNA sequence analysis
	XM_538911.3	5UTRcADCY10	F: 5'-AATGTTTTTGCTGGGTTGTAGCC-3'	cDNA cloning
		3UTRcADCY10	R: 5'-AATCCTTCTCCGTAGAGCAGCAGA-3'	cDNA cloning
Canine *TREML1*	NC 006594.3	TREML1ex1F	F: 5'-GGCTAGCTGGATGCACAGAGCTTTGG-3'	Genomic DNA sequence analysis
		TREML1ex1R	R: 5'-GCTTCCTCCCAAAGGGGACTGGGGC-3'	Genomic DNA sequence analysis
		TREML1ex23F	F: 5'-CTCATCCTGGGGCTTCTCAGTGAAC-3'	Genomic DNA sequence analysis
		TREML1ex23R	R: 5'-CTGAACCTGGCAGGAAGCTGCTC-3'	Genomic DNA sequence analysis
			F: 5'-GTTAGCCCGGGGCTCTGAACAATC-3'	Genomic DNA sequence analysis
		TREML1ex4R	R: 5'-CTCTCCCATCCCTCAGATCTCAGGC-3'	Genomic DNA sequence analysis
		TREML1ex56F	F: 5'-CCAAAGACCCAAGAGGCAGCTG-3'	Genomic DNA sequence analysis
		TREML1ex56R	R: 5'-CTGGGGAACGCTGTGTCAGTGGCC-3'	Genomic DNA sequence analysis
	XM_532133.3	5UTRcTREML1	F: 5'-CTGACATCTGCCTGACCTGCC-3'	cDNA cloning
		3UTRCTREML1	R: 5'-GTGAGTGTACAGTATGATGAACAGC-3'	cDNA cloning
Canine *TREM2*	NC_006594.3	TREM2ex1F	F: 5'-GAGATCTTGCACAAGGTACCCAGTG-3'	Genomic DNA sequence analysis
		TREM2ex1R	R: 5'-CCACCCACCTGCCGCCATATACTC-3'	Genomic DNA sequence analysis
		TREM2ex2F	F: 5'-CGTTCATTGCACACCGTAAGTGTTCG-3'	Genomic DNA sequence analysis
		TREM2ex2R	R: 5'-GCACAGACATCAGAGACATAGTCCCAG-3'	Genomic DNA sequence analysis
Canine *TREM2*	NC_006594.3	TREM2ex34F	F: 5'-CCAGCTGCCTGAAGGAGGAGGGAC-3'	Genomic DNA sequence analysis
		TREM2ex34R	R: 5'-GATCTTGTGCTCACATGGCGCCTCTGC-3'	Genomic DNA sequence analysis
		TREM2ex5F	F: 5'-CACCCCAAGTCGCCAGCAGAGCCTG-3'	Genomic DNA sequence analysis
		TREM2ex5R	R: 5'-CTCCCCTCCCACTAGTCCATGGCTC-3'	Genomic DNA sequence analysis
		TREM2ex6F	F: 5'-CCTGTGACCAGGGCCACATGCAGCTGC-3'	Genomic DNA sequence analysis
		TREM2ex6R	R: 5'-CATCCCAGTGGTGATCTGTACACTGC-3'	Genomic DNA sequence analysis

Genes	GenBank Accession numbers	Primer ID	Nucleotide sequences	Purposes
	XM _846138.2	5UTRcTREM2	F: 5'-GCCCTCTGCAAGGGAAACCCGAC-3'	cDNA cloning
		3UTRcTREM2	R: 5'-CTACAGGTGTGCTCCAGTGCCGC-3'	cDNA cloning
Canine *CMRF35L5*	NC_006594.3	CMRF35L5ex12F	F: 5'-CTGGCTCCCTGTCCTCAGGTCTTCCC-3'	Genomic DNA sequence analysis
		CMRF35L5ex12R	R: 5'-GCATCTTGTGATTCAGGATGGCTGAG-3'	Genomic DNA sequence analysis
		CMRF35L5ex34F	F: 5'-CTGGAATGTGGGGCAAACACATGG-3'	Genomic DNA sequence analysis
		CMRF35L5ex34R	R: 5'-ATGAGGACATCATGGCCAAGCCCTC-3'	Genomic DNA sequence analysis
		CMRF35L5ex56F	F: 5'-CTTCCCAGGGTAACGGGACCTATGG-3'	Genomic DNA sequence analysis
		CMRF35L5ex56R	R: 5'-CTGGTGGAGCAGCCTTATGCCTCAC-3'	Genomic DNA sequence analysis
	XM_846150.2	5UTRcCMRF35L5	F: 5'-GCCACGCTCAAGAAGAGGGCC-3'	cDNA cloning
		3UTRcCMRF35L5	R: 5'-GTTCACTTCCCAGTGAAGACTGAC-3'	cDNA cloning
Canine *TREML2*	NC_006594.3	TREML2ex1F	F: 5'-GGCGTGGGGCCCAGACCCTGGATG-3'	Genomic DNA sequence analysis
		TREML2ex1R	R: 5'-GAAAAGCCTGGAGGGAGACTCCAG-3'	Genomic DNA sequence analysis
		TREML2ex2F	F: 5'-CTCACCTCAACCTTCAAGATGCCTCC-3'	Genomic DNA sequence analysis
		TREML2ex2R	R: 5'-CTGCTGCGAATGAAGTATGAGGCAG-3'	Genomic DNA sequence analysis
		TREML2ex3F	F: 5'-CTGCTTCTATCGGGGAGGCCTCTGAC-3'	Genomic DNA sequence analysis
		TREML2ex3R	R: 5'-GTTCAGGCACCAAGAACTGTTGCTG-3'	Genomic DNA sequence analysis
	XM_538912.3	5UTRcTREML2	F: 5'-CCGTCCCCAACTGGATGGAGCC-3'	cDNA cloning
		3UTRcTREML2	R: 5'-ACTCCACAGTTAAGTGGTCTCAG-3'	cDNA cloning
Canine *TREM1*	NC_006594.3	TREM1ex1F	F: 5'-GCTTCGAGGTTAGAGTGTCCCAGG3'	Genomic DNA sequence analysis
		TREM1ex1R	R: 5'-GCTCTATGCTGGGTAGCCACATCC-3'	Genomic DNA sequence analysis
		TREM1exF3	F: 5'-GAGTGTCCCAGGCACATCAGTGC-3'	Genomic DNA sequence analysis
		TREM1ex2F	F: 5'-CCAAAGAGAGGAAAACTATTCCTC-3'	Genomic DNA sequence analysis
		TREM1ex2R	R: 5'-GCCCATACCCTCACCTGGCCTGGC-3'	Genomic DNA sequence analysis
		TREM1ex3F	F: 5'-CTAATGGCTGAGTAATATTCCATGC-3'	Genomic DNA sequence analysis
		TREM1ex3R	R: 5'-CATTCCCCACAGCTTCTCAAGCAC-3'	Genomic DNA sequence analysis
		TREM1ex4F	F: 5'-GAGAAGGGGCATGAAGGACTTCTG-3'	Genomic DNA sequence analysis
		TREM1ex4R	R: 5'-CAGCTCTCCTCCAGGACAATTGCC-3'	Genomic DNA sequence analysis
	XM 846172.2	5UTRcTREM1	F: 5'-CTGGTGTTGGCGCACATGAAGG-3'	cDNA cloning
		3UTRcTREM1	R:5'-GGTCATTCTCATGGCTTCACCAGC-3'	cDNA cloning
Canine *TSPO2*	XP_022281477.1	5UTRcTSPO2	F: 5'-AGATTTTGCCTCTGTGAGGGGA-3'	PCR-RFLP of canine TSPO2 C40Y mutation
		cTSPO2int1r	R: 5'-GCCGTGGTATGAGAAGTGGGTGC-3'	PCR-RFLP of canine TSPO2 C40Y mutation
		cTSPO2ex2f	F: 5'-GCCCTGCCACCATCTCTCCACAG-3'	PCR of canine TSPO2 VFT mutation
		cTSPO2F3	R: 5'-ATGGGCGTGGGCTGCAAAGAAGAAG-3'	PCR of canine TSPO2 VFT mutation
		cTSPO2dF98	R: 5'-ATGGGCGTGGGCTGCAAAGAAGATC-3'	PCR of canine TSPO2 VFT mutation

Genes	GenBank Accession numbers	Primer ID	Nucleotide sequences	Purposes
		cTSPO2qp1	F: 5'-CCTCACAAAGTCTTGTTGGC-3'	qRT-PCR
		cTSPO2qp2	R: 5'-TCCCAGGTCCTTCCATACC-3'	qRT-PCR
Canine *GAPDH*	NM_001003142.1	cGAPDHp1	F: 5'-TGCTCCTTCTGCTGATGCCCCCAT-3'	qRT-PCR
		cGAPDHp2	R: 5'-TCTGGGTGGCAGTGATGGCATGGA-3'	qRT-PCR
Human *TSPO2*	NM_001010873.3	hTSPO2qp1	F: 5'-AACAGGGCAGCCAGTTTTGTTGATG-3'	qRT-PCR
		hTSPO2qp2	R: 5'-AGCTGGACTGTCCTGGTTCTCTTT-3'	q-RT-PCR
Mouse *Gapdh*	NM_001289726	mouse GAPDH-RT-F	F: 5'- GTCTTCACCACCATGGAGAAG -3'	qRT-PCR
		mouse GAPDH-RT-R	R: 5'-GCCATCCACAGTCTTCTGGGT-3'	qRT-PCR
Mouse *Tspo2*	NM_027292.2	mTSPO2-RT-F	F: 5'-ACCGACTGGATGGCTGATGAATGA-3'	qRT-PCR
		mTSPO2-RT-R	R: 5'-ATTGGGCAGACCAACCTGGATCTT-3'	qRT-PCR
Mouse *α-globin*	NM_008218.2	mouse α-globin-RT-F	F: 5'-CTCTCTGGGGAAGACAAAAGC-3'	qRT-PCR
		mouse α-globin-RT-R	R: 5'-GGTGGCTAGCCAAGGTCACCA-3'	qRT-PCR
Mouse *β-globin*	NM_001278161.1	mouse β-globin-RT-F	F: 5'-TTCTGACATAGTTGTGTTGACTCAC-3'	qRT-PCR
		mouse β-globin-RT-R	R: 5'-TCGGAGTTCACCTTTCCCCA-3'	qRT-PCR
Mouse *Delta-aminolevulinate synthase 1*	NM_001291835.1	mouse ALAS1-RT-F	F: 5'-CGAGTCACATCATCCCTGTG-3'	qRT-PCR
		mouse ALAS1-RT-R	R: 5'-TTAAGTTCCAGCCCAACTCG-3'	qRT-PCR
Mouse *Delta-aminolevulinate synthase 2*	NM_009653.3	mouse ALAS2-RT-F	F: 5'-ATCTGTGCGCCTACTCAAGG-3'	qRT-PCR
		mouse ALAS2-RT-R	R: 5'-TGTGCTTGGAGAGCAGAAGA-3'	qRT-PCR
Mouse *GATA binding protein 1*	NM_008089.2	mouse GATA1-RT-F	F: 5'-CAGAACCGGCCTCTCATCC-3'	qRT-PCR
		mouse GATA1-RT-R	R: 5'-TAGTGCATTGGGTGCCTGC-3'	qRT-PCR
Mouse *GATA binding protein 2*	NM_008090.5	mouse GATA2-RT-F	F: 5'-GAATGGACAGAACCGGCC-3'	qRT-PCR
		mouse GATA2-RT-R	R: 5'-AGGTGGTGGTTGTCGTCT-3'	qRT-PCR
Mouse *anion exchanger 1*	NM_011403.2	mouse AE1-RT-F	F: 5'-TATGGGGTCGCCCACATCTAT-3'	qRT-PCR
		mouse AE1-RT-R	R: 5'-AGGCCGAATCTGATCCTCGTA-3'	qRT-PCR
Mouse *Kruppel-like factor 1*	NM_010635.3	mouse KLF1-F	F: 5'-AGAGTGGATCCAAGGACCGT-3'	qRT-PCR
		mouse KLF1-R	R: 5'-CCTCTGGTCTAGGGGTCCAT-3'	qRT-PCR
Mouse *Setd8*	NM_030241.3	mouse Setd8-F	F: 5'-CAGACCAAACTGCACGACATC-3'	qRT-PCR
		mouse Setd8-R	R: 5'-CTTGCTTCGGTCCCCATAGT-3'	qRT-PCR
Mouse *Tspo2*	NM_027292.2	mTSPO2 ex1	F: 5'-ATGCAGCTTCAAGGACCTGTCTT-3'	Genomic DNA sequence analysis
		mTSPO2ex1anti	R: 5'-CCATGACAGAGTAGATGGTCACC-3'	Genomic DNA sequence analysis

Genes	GenBank Accession numbers	Primer ID	Nucleotide sequences	Purposes
Mouse *p16*	NM_001040654.1	mouse p16-F	F: 5'-TTTCGTGAACATGTTGTTGAGGCTA-3'	qRT-PCR
		mouse p16-R	R: 5'-GCTACGTGAACGTTGCCCATC-3'	qRT-PCR
Mouse *p53*	NM_001127233.1	mouse p53-F	F: 5'-GTATTTCACCCTCAAGATCC-3'	qRT-PCR
		mouse p53-R	R: 5'-TGGGCATCCTTTAACTCTA-3'	qRT-PCR
Mouse *Fas*	NM_001146708.1	mouse fas-F	F: 5'-TGCTTGCTGGCTCACAGTTA-3'	qRT-PCR
		mouse fas-R	R: 5'-CAGCAAAATGGGCCTCCTTG-3'	q-RT-PCR
Mouse *Fas ligand*	NM_001205243.1	mouse fasl-F	F: 5'-TCCATCTTGTGGGCCTAGAG-3'	qRT-PCR
		mouse fasl-R	R: 5'-TCCTAATCCCATTCCAACCA-3'	qRT-PCR
Mouse *Trail*	NM_009425.2	mouse trail-F	F: 5'-CTTCCGATTTCAGGAAGCTG-3'	qRT-PCR
		mouse trail-R	R: 5'-GGATCCGGATAGCTGGTGTA-3'	qRT-PCR

Supplemental Figures and Legends

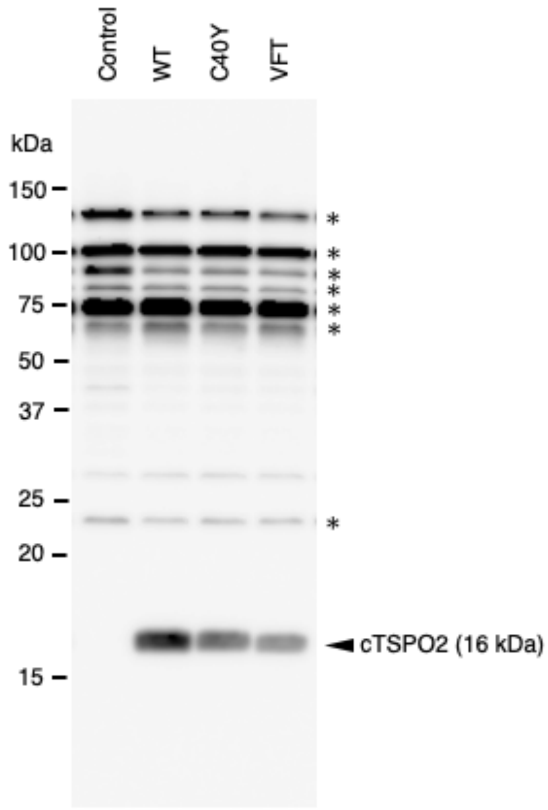

Figure S1. Immunoblot analysis of cTSPO2 in stably transfected K562 cells

Proteins were extracted from K562 cells stably expressing the wild-type (*WT*), C40Y (*C40Y*), and VFT (*VFT*) cTSPO2 as well as from the empty vector-transfected control cells (Control) as described previously (49). cTSPO2 was detected by immunoblotting using the anti-cTSPO2 antibody. In addition to the bands of 16-kDa cTSPO2 monomer, several bands with the sizes of 23~130 kDa in the immunoblots were observed sometimes in all cell lines including control cells (indicated by asterisks) and appeared to be some unidentified polypeptides nonspecifically reacting to the antibodies. Migrating positions of marker proteins are shown in kDa.

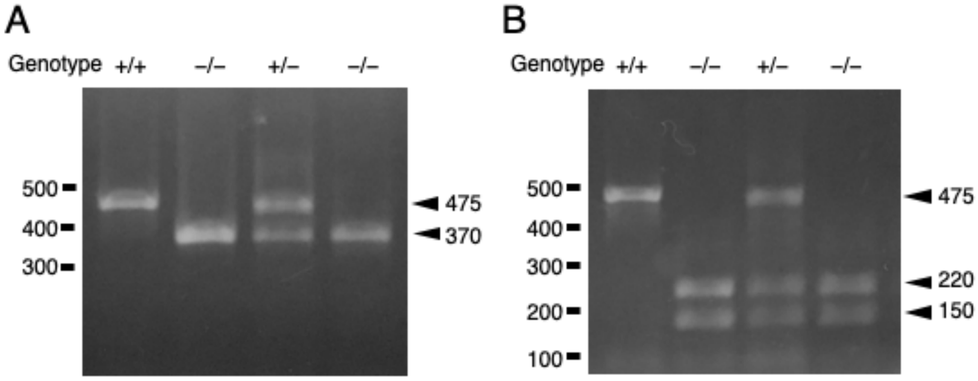

Figure S2. Genotyping of *Tspo2* knockout mice

The *Tspo2* knockout mice with the Δ105 mutation were genotyped by PCR followed by digestion of the PCR products with *Pst* I. PCR amplification of genomic DNA generated 475-bp and 370-bp fragments from the wild-type and Δ105 mutant alleles, respectively, so that we could identify the genotypes for *Tspo2* as the wild-type (+/+) and heterozygous (+/–) and homozygous (–/–) for the Δ105 mutation (A). The Δ105 mutation was confirmed by digestion with *Pst* I, since the Δ105 mutation created a *Pst* I restriction site in the mutated allele. The 475-bp fragment remained intact, while the mutant allele-derived 370-bp fragment was digested into two fragments (220 and 150 bp) (B). Migrating positions of the size marker DNA are shown in bp at left sides.

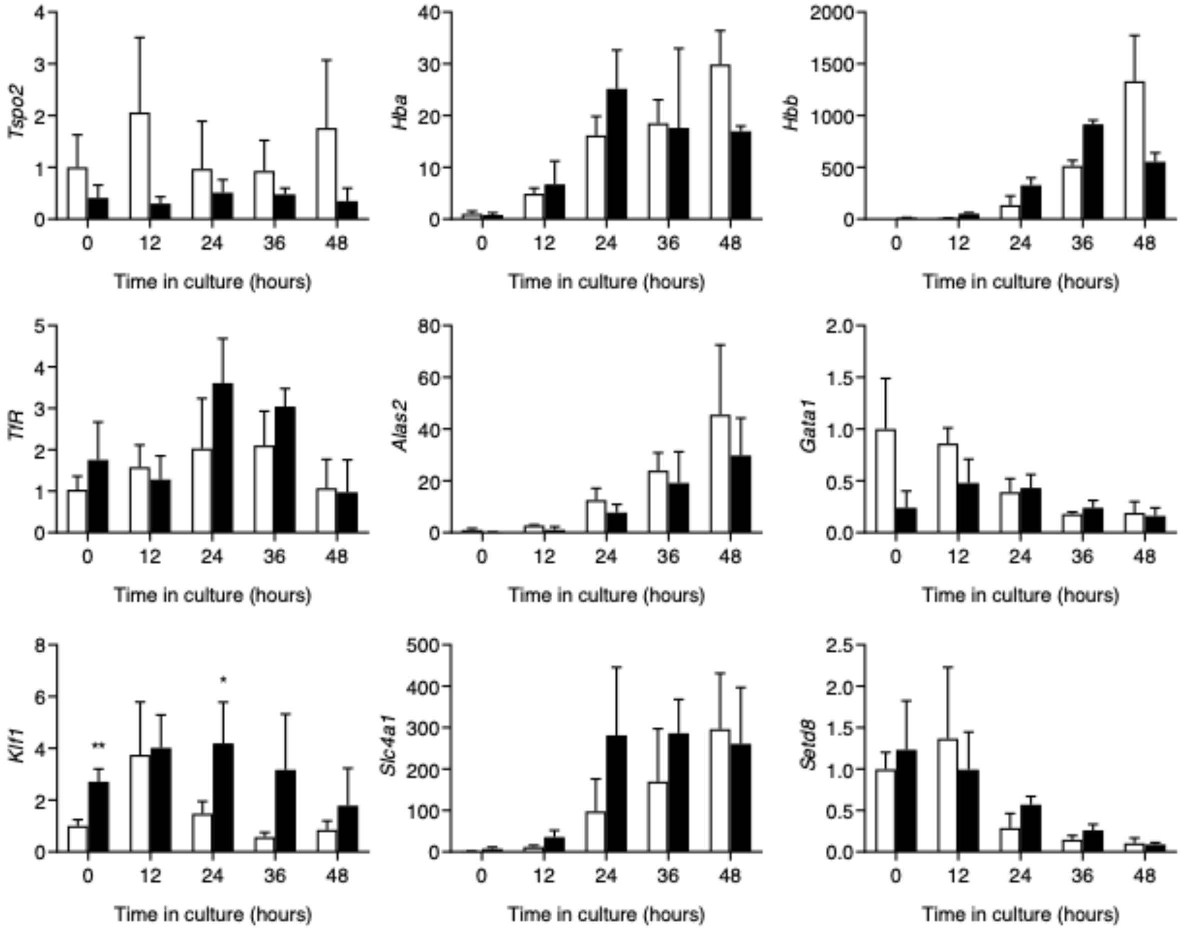

Figure S3. Expression of erythroid-specific genes in maturing MEDEP cells

The relative abundance of mRNAs of some late erythroblast-specific genes indicated, *Tspo2*, *Hba* (α-globin), *Hbb* (β-globin), *TfR* (transferrin receptor), *Alas2* (5'-aminolevulinate synthase 2), *Gata1*, *Klf1/Eklf*, *Slc4a1* (anion exchanger 1, band 3), and *Setd8* (histone methyltransferase), were analyzed by qRT-PCR. The data are normalized by the expression level of *Gapdh* and expressed as the mean ± S.D. (n = 3) relative to that in control MEDEP cells at time 0. *Tspo2* mRNA levels in *Tspo2*$^{-/-}$ cells (indicated by black bars) were lower than those in control cells (indicated by open bars) throughout the incubation period. There were no major differences between control and *Tspo2*$^{-/-}$ cells in mRNA expressions of other genes through incubation period except that higher expression of *Klf1* was apparent in *Tspo2*$^{-/-}$ cells at several time points.

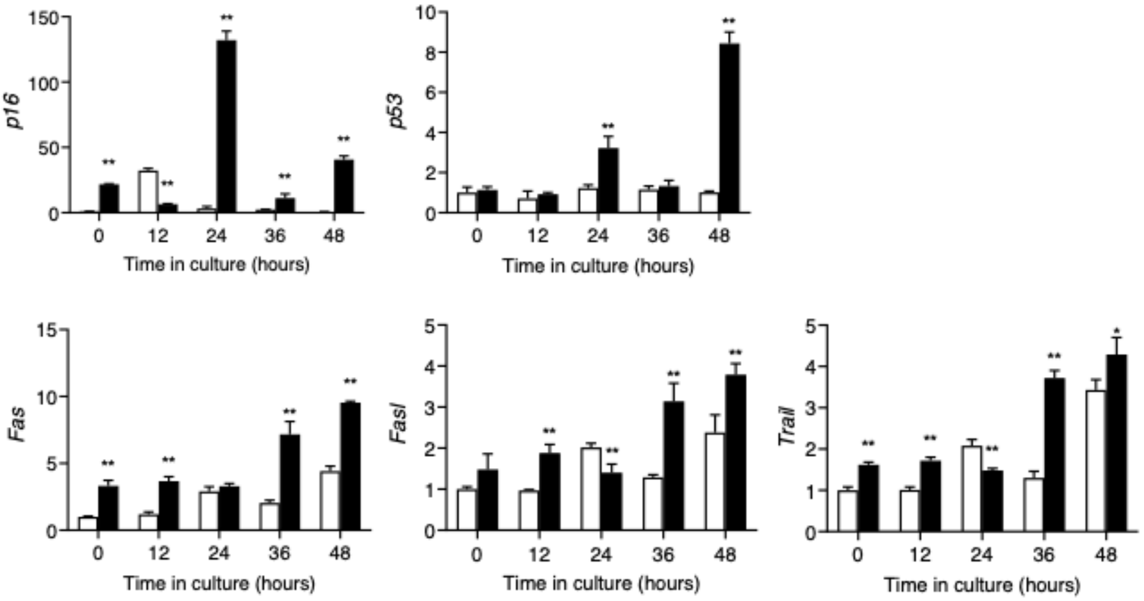

Figure S4. Expression of cell-cycle- and apoptosis-related genes in maturing MEDEP cells

The relative abundance of mRNAs of some cell cycle- and apoptosis-related genes indicated, *p16*, *p53*, *Fas*, *Fasl* (Fas-ligand), and *Trail* (TNF-related apoptosis-inducing ligand), were analyzed by qRT-PCR. The data are normalized by the expression level of *Gapdh* and expressed as the mean ± S.D. (n = 3) relative to that in control MEDEP cells at time 0. The mRNA levels of these genes in *Tspo2*$^{-/-}$ cells (indicated by black bars) were higher than those in control cells (indicated by open bars) throughout the incubation period with several exceptions.
